# New Regional Dynamic Cancer Model across the European Union

**DOI:** 10.3390/cancers15092545

**Published:** 2023-04-28

**Authors:** Silvius Ioan Negoita, Romeo Victor Ionescu, Monica Laura Zlati, Valentin Marian Antohi, Alexandru Nechifor

**Affiliations:** 1 Anaesthesia Intensive Care Unit, Department Orthopedics, University of Medicine and Pharmacy Carol Davila of Bucharest, 020021 Bucharest, Romania; 2 Department of Administrative Sciences and Regional Studies, Dunarea de Jos University of Galati, 800008 Galati, Romania; 3 Department of Business Administration, Dunarea de Jos University of Galati, 800008 Galati, Romania; 4 Departament of Finance, Accounting and Economic Theory, Transilvania University of Brasov, 500036 Galati, Romania; 5 Department of Medical Clinical, Dunarea de Jos University of Galati, 800008 Galati, Romania

**Keywords:** cancers, economic welfare, regional dynamic cancer model, regional cancer disparities

## Abstract

**Simple Summary:**

At the European level, the incidence and mortality rates of various types of cancer have been increasing since the 1990s (after the fall of the former Communist Bloc). EU member states have had to allocate substantial funds to fight and treat cancers, but the effects are far from what were expected and there is an increased awareness of the disease among the population. The numerous studies carried out in the literature show the interest of researchers in this field and support our present scientific approach. Our study aimed to conceptualise a dynamic regional cancer model projected at the European level for the period 1993–2021, showing the impact of increasing economic welfare and financial allocations to health on disease incidence and mortality rates. The results of the study demonstrate that there are significant differences in these influences on the male and female population, which are also influenced by the type of cancer.

**Abstract:**

Background: Can increasing levels of economic wealth significantly influence changes in cancer incidence and mortality rates? Methods: We investigated this issue by means of regression analyses based on the study of incidence and mortality indicators for lip, oral cavity, and pharyngeal; colon; pancreatic; lung; leukaemia; brain and central nervous system cancers in correlation with the levels of economic welfare and financial allocations to health at the level of the European Union member states, with the exception of Luxembourg and Cyprus for which there are no official statistical data reported. Results: The results of the study showed that there were significant disparities both regionally and by gender, requiring corrective public policy measures that were formulated in this study. Conclusions: The conclusions highlight the main findings of the study in terms of the evolution of the disease, present the significant aspects that characterise the evolution of each type of cancer during the period analysed (1993–2021), and highlight the novelty and limitations of the study and future directions of research. As a result, increasing economic welfare is a potential factor in halting the effects of cancer incidence and mortality at the population level, while the financial allocations to health of EU member countries’ budgets are a drawback due to large regional disparities.

## 1. Introduction

**Figure 1:**
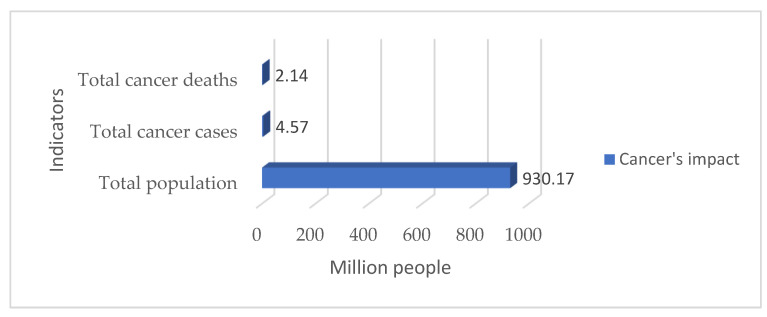
Impact of cancer on the European population (million people, 2018). Source: World Health Organization [1].

Cancer is a scourge with which humanity continues to struggle. The latest official statistics state that there were more than 4.5 million cancer cases and more than 2.1 million cancer deaths in Europe in 2018 [1] (see Figure 1).

**Figure 2:**
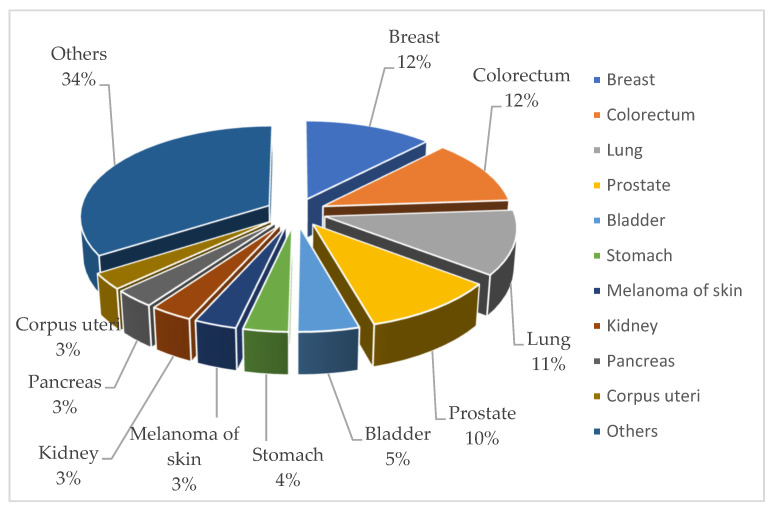
Cancer incidence in the European population (2018). Source: World Health Organization, Burden of Cancer [1].

Cancer incidence rates range from 2.9% (corpus uteri) to 12.3% (breast), with breast, colorectal, lung, and prostate cancers having the highest incidence rates (see Figure 2).

**Figure 3:**
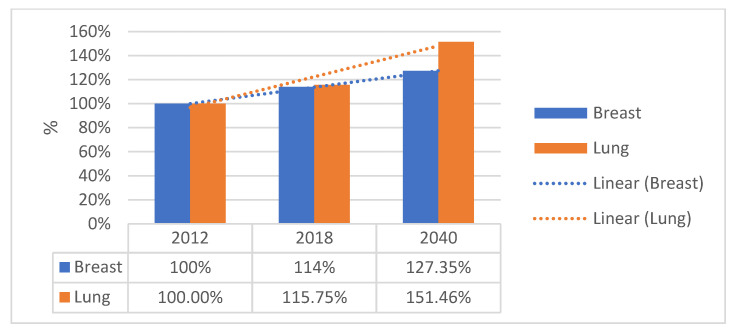
Trends in total cases of breast and lung cancers at the European population level (2012 = 100%). Source: World Health Organization, Burden of Cancer [1].

Cancer will continue to be a major challenge at the European level. Breast and lung cancer incidence projections to 2040 predict a significant increase in annual cases (see Figure 3).

Implementing a package of essential services for cancer patients will result in increased additional costs per patient from $1.60 in 2020, to $3 in 2026, and $4 in 2030.

The novelty of the present scientific approach lies in the integrated multi-criteria approach to a broad range of cancer symptoms in relation to the evolution of population welfare and the level of funding of the public health system. The analysis covers 25 EU member states for which official statistical data are available. Another novel element is the conceptualisation of the regional dynamic cancer model, the outputs of which will highlight the evolution of the disease in terms of incidence and mortality rates in the context of achieving a certain economic performance.

Given the increasing incidence and mortality of cancers in the population and in the context of the recent threats posed by epidemiological health crises, it is necessary to structure a model of the evolution of the disease in relation to the financial allocations to health and the possibility of the population accessing the latest treatments in the field. This aspect motivates and supports the present research, with the following objectives:

O1. Study trends in incidence and mortality rates for the analysed cancers (lip, oral cavity, and pharyngeal; colon; pancreatic; lung; leukaemia; brain and central nervous system).

O2. Reflect on new trends in the literature on how to diagnose and treat these cancers in order to limit the increase in incidence and mortality rates.

O3. Conceptualise the regional dynamic cancer model, and conduct testing and validation of the model.

O4. Stage the dynamic regional pattern of cancer by sex and compare the effects of increasing economic welfare on the incidence and mortality rates of the six types of cancer.

The study continues with a literature review, presentation of the used materials and working methods, definition of research hypotheses, presentation of results, discussion, and conclusions.

## 2. Literature Review

**Figure 4:**
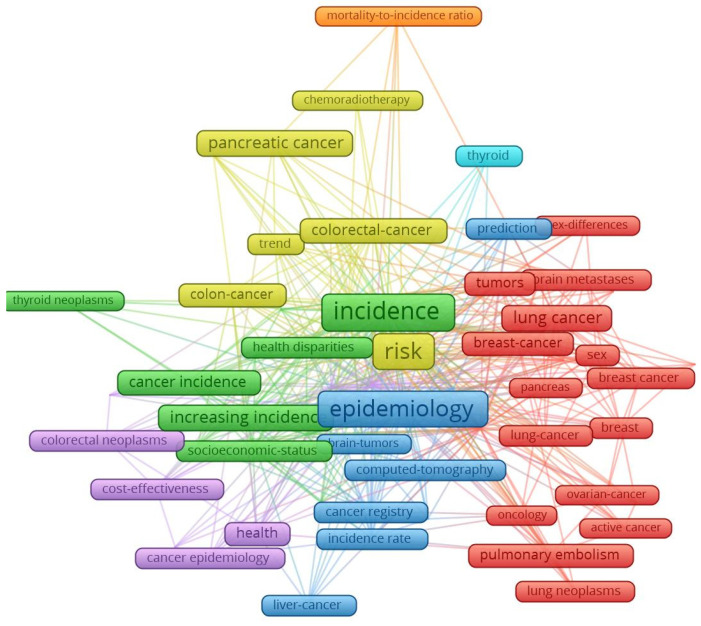
Areas of interest in cancer research in the literature.

In the literature, this topic has received a great deal of attention, given the major impact on the health status of the population and the high costs of specific treatments. A summary of the flow of publications in the field is shown in Figure 4, based on a selection of 9559 articles published in the period 2018–2022 in the areas of interest: lung cancer (1199 articles), thyroid dysfunctions and neoplasms (1213 articles), colon and liver cancers (1111 articles), incidence of pancreatic cancers (1151 articles), incidence of brain cancers (1113 articles), and blood diseases and leukaemias (3773 articles).

Figure 4 was realised by the authors using the Web of Science platform and VOSViewer software. The analysis of the scientific interests in cancer researchrevealed that the 9559 articles were cited more than 104,000 times in approximately 68,000 scientific publications, with an average of 11 citations per item and a Hirsh index calculated on the Web of Science platform of 113 points. It can be seen that interest is growing in the dynamics of cancer, and interest in pancreatic and hepatocellular cancers are top areas accumulating over 1000 citations per article.

The main areas of research on which experts have conducted studies focus on disease trends, risk factors, prevention strategies, mortality and survival rates, clinical diagnostics, and therapeutic strategies.

A regional analysis for cancers associated with modifiable risk factors was performed by Cabasag et al. [2]. The analysis covered 38 European countries and looked at cancer incidence by age group based on information from the GLOBOCAN database. The authors calculated specific incidence rates for each state in the sample according to cancer location, sex, and age group. The results of the analysis showed that 33% of cancers in men and 44% in women were potentially preventable. The most common cancers were lung, colorectal, female breast, laryngeal, and oesophageal cancers, but regional disparities across Europe were extremely wide. The authors proposed additional population-based prevention measures to reduce modifiable risk factors for cancer. The global approach to cancer cases was the subject of research by Sung et al. [3]. Based on the study of information provided by the International Agency for Research on Cancer, the authors stated that there were 19.3 million new cancer cases and 10.0 million cancer deaths in 2020. In terms of incidence, breast cancer in women was the most common, followed by lung, colorectal, prostate, and stomach cancers. In terms of death rates, lung cancer ranked first, followed by colorectal, liver, stomach, and female breast cancers. Global cancer incidence was directly influenced by the level of socio-economic development. The authors’ predictions of global cancer incidence in 2040 were extremely worrying. The GLOBOCAN 2020 database was used by Ferlay et al. [4] to highlight cancer incidence and mortality by age group and sex in 185 countries or territories worldwide. Following the analysis, the authors found that the most common cancers worldwide in 2020 were female breast, lung, and prostate cancers. The highest mortality rates were due to lung, liver, and stomach cancers. Authors such as Soerjomataram & Bray [5] estimated that global cancer incidence would double in 2070 compared to 2020. The authors started their analysis from risk factors such as tobacco, overweight, obesity, and human papillomavirus infection, concluding that the world’s countries needed to implement national cancer control programmes at the highest level. In the US, the National Center for Health Statistics monitors cancer occurrence and outcomes in the population. According to a study by Siegel et al. [6], the leading cause of death was lung cancer, followed by breast and prostate cancers. The authors concluded that further investment in cancer control and improved early detection and treatment would support a significant decrease in cancer mortality. Global cancer incidence and mortality rates among children aged 0–14 years were reviewed by Huang et al. [7]. The authors related this data to each country’s human development index, reporting that the highest childhood cancer mortality rates were found in low-income countries. At the European level, the main cancer sites were analysed by Dalmartello et al. [8] over the period 1989 to 2022. The authors looked at 10 cancer sites, target cancer mortality rates, and absolute numbers of deaths obtained from the World Health Organization and Eurostat databases. The analysis showed that the number of deaths due to lung cancer had decreased in both men and women. Deaths from stomach, colorectal, breast, and prostate cancers also decreased over the period. Deaths due to pancreatic cancer remained stable in men and increased in women, while those due to bladder cancer decreased in men but remained stable in women. Leukaemia and ovarian cancer mortality had decreased in all countries considered. A new global cancer analysis (204 countries and territories) was conducted by [9] based on data from the Global Burden of Diseases, Injuries, and Risk Factors Study 2019. The authors covered the period 2010–2019 and used indicators of cancer incidence, morbidity, and mortality. The analysis found that cancer was second only to cardiovascular disease in the number of deaths and years of life lost globally in 2019. Globally, estimates of the costs and benefits of investing in improved cancer survival are few, as claimed by Ward et al. [10]. The authors examined the lifetime health, economic costs, and benefits of disease treatment packages. The study covered 200 states and 11 cancers (esophageal, stomach, colon, rectum, anus, liver, pancreatic, lung, breast, cervix, and prostate cancers) and concluded with a 2030 forecast of the evolution of these cancers. It was found that without significant progress in identifying and treating the 11 cancer, there would be 76 million cancer deaths globally for patients diagnosed between 2020 and 2030. According to [11], the American Cancer Society annually evaluates incidence data collected by central cancer registries and mortality data collected by the National Center for Health Statistics. A higher incidence was seen for prostate cancer, but overall incidence trends were more favourable for men than women. For men, the incidence of liver cancer and melanoma had stabilised in those aged over 50 years and decreased in younger men. For women, the incidence of lung and cervical cancers had decreased and the incidence of breast, uterine, and liver cancers and melanoma had increased. Death rates due to leukaemia, melanoma, lung, and kidney cancers had continued to fall amid advances in treatment. The level of economic development and well-being of the US population supported the implementation of effective cancer treatments with beneficial effects on incidence and death rates in this country. We used a similar approach in this paper, which led to similar results for incidence and mortality rates. According to Miller et al. [12], the most common cancers in the US were prostate, melanoma of the skin, colon, and rectum cancers among men and breast, corpus uteri, and thyroid cancers among women. The authors noted the disparities that existed between the black population and the majority of the population’s access to cancer investigation and treatment techniques. Therefore, the authors proposed implementing new strategies to mitigate disparities for communities of colour and optimise care for people with a history of cancer. The correlations between health service planning, resource management, and prediction of cancer incidence and mortality were the subject of a study by Luo et al. [13]. The analysis covered 21 individual cancers in Australia and looked to 2044. The modelling used an age cohort model and a generalised linear model for each cancer type. The modelling showed that the incidence for all cancers combined decreased for men and became relatively stable for women over the forecasted horizon. In the case of mortality rates for all cancers combined, they trended downwards for both men and women. To counter the effects of cancer, the authors proposed the extensive use of screening and control of risk factors (smoking, obesity, physical inactivity, alcohol consumption, and infections). A comparative analysis of the impact of cancer on society looking at the US and China was conducted by Changfa et al. [14]. The analysis used the specialised GLOBOCAN 2020 database and population estimates from the United Nations. It found that breast cancer was the most common cancer in the US, while lung cancer was predominant in China. On the other hand, lung cancer was the leading cause of cancer deaths in both countries analysed. The analysis showed that the US and China had converging cancer profiles, characterised by declining incidence and mortality rates for liver, stomach, and oesophageal cancers and increasing rates for lung, colorectal, breast, and prostate cancers. The need to predict cancer incidence and mortality rates in direct correlation with achieving effective and inclusive public health systems was supported by Tudor [15]. The analysis covered Romania, which consistently reported higher mortality rates from all cancers than the EU27 average. The authors believed that this phenomenon was due to inefficiencies in the public health system and underfunding, which supported the development of the “onco-tourism” phenomenon. As a result, a forecast of cancer incidence and mortality rates in Romania was made based on a questionnaire sent to a net sample of the population. Multiple statistical and econometric methods were used to process the statistical data obtained (ARIMA, exponential smoothing state–space model with Box-Cox transformation, TBATS, and an autoregressive nonlinear feed-forward neural network model). The modelling results showed a continuation of the increasing trends in cancer incidence and mortality in Romania until 2026, assuming no significant changes in the public health system. Data taken from the American Cancer Society on breast cancer in women were used by Giaquinto et al. [16], who focused on the increase in incidence rates over the last 40 years but also on the decrease in mortality rates over the same time frame. The authors believed that the optimal solutions to more drastically decrease the mortality rate of this cancer would be to increase access to high-quality health care, high-quality screening, and develop partnerships between public and private health systems and patient cohorts. Another analysis looking at breast cancer incidence and mortality was performed by Chenyu et al. [17] and covered 60 countries of the world over the period 2000–2019. The authors used Joinpoint analysis and multivariate linear regression, where incidence and mortality rates were “coupled” with statistical data on people’s behaviour and socio-economic development. Higher incidence and mortality rates were observed in countries with a higher human development index. Prostate cancer incidence and mortality were analysed by Wang et al. [18] using the GLOBOCAN 2020 database. The analysis covered 174 countries over the period 2000–2019 and highlighted correlations between prostate cancer incidence and mortality rates and human development indices in the countries analysed. The overall trend was an increase in incidence and a reduction in mortality due to prostate cancer in countries with a positive human development index trend. In the opinion of Hossain et al. [19], colorectal cancer was the second most lethal cancer, as the incidence and mortality of this type of cancer showed increasing trends worldwide. Death rates were high in developed countries, but there had been a significant increase in developing countries. Lung cancer is the leading cause of cancer deaths worldwide. An analysis based on GLOBOCAN 2020 for 21 regions and 185 countries was carried out by (15, 2022), focusing on the Mortality-Incidence Ratio (MIR). The authors also made a forecast for the year 2050, theorising that the incidence and mortality rates of this type of cancer would each be higher than 3.2. Correlations between incidence and mortality rates with smoking prevalence and the regional human development index were also highlighted. Another study looking at lung cancer incidence and mortality was conducted by Huang et al. [20] based on correlations with the human development index, gross domestic product (GDP), and smoking prevalence. Lung cancer incidence and mortality trended upwards in women and downwards in men.

Cancer incidence and mortality rates in Africa were studied by Sharma et al. [21] in regional profile and taking into account the economic burden that this disease presents in the context of macroeconomic development. The authors also forecasted the impact of cancer on the African economy and society by 2040, using incidence and mortality indicators by age, sex, and country. Using linear regression and taking into account the human development index, maps were generated for each cancer group for each country in Africa. The analysis concluded that reducing cancer incidence and mortality in Africa could only be achieved under conditions of economic growth that would allow for the financing of a holistic approach to cancer control and management.

Declining cancer mortality rates in Europe over the past three decades was the finding of Dalmartello et al. [8]. The analysis covered the period 1989–2022 for the European Union, the United Kingdom, France, Germany, Italy, Poland, and Spain. Mortality rates for lung, stomach, colorectal, breast, prostate, pancreatic, uterine, bladder and leukaemia cancers were discussed separately for men and women.

The impact of cancer at the European level was the focus of the study by Dyba et al. [22], who analysed the incidence and mortality for 25 major cancers in 40 European countries, including the EU27, for the year 2020. The authors found that the most common cancers in Europe were female breast, colorectal, lung, and prostate cancers. Lung cancer was the leading cause of death, followed by colorectal, breast, and pancreatic cancers. Finally, the authors proposed prioritising cancer control across Europe. An EU-wide study realised by Belot & Pohar-Perme [23] highlighted the disparities that existed between European citizens in relation to the widening gap between health progress and health equity. In the field of cancer, the authors stated that there are 200 diseases, which requires a systems approach to understanding cancer. In addition, disparities in the approach, treatment, and means available to European citizens in the fight against cancer were still extremely wide.

Two specific cancers (oral cavity and oropharyngeal cancers) were analysed in Spain over the period 1979–2018 in relation to mortality rates. The statistical rates were standardised by the authors by age group and modelled using regression analysis to quantify the impact of age, period, and cohort on mortality rates for the two cancers. The authors believed that the decrease in mortality rates was due to a reduction in tobacco/alcohol consumption in conjunction with careful monitoring of these cancers by age group, sex, and location as well as continued preventive treatment against known risk factors. The same topic was addressed by Sofi-Mahmudi et al. [24], who conducted an analysis of lip and oral cavity cancer worldwide using the Global Burden of Disease (GBD) Study database. The authors defined four main indices of quality of care for lip and oral cavity cancer (incidence, years of life lost, years lived with disability, and disability-adjusted life years) and used principal component analysis (PCA) to quantify a quality of care index in this area. The analysis revealed that the quality of care was on an upward trend over the period 1990–2017. In this context, the highest index values were recorded in North America, Western Europe, and Australia. At the other end of the spectrum were Central Africa and Afghanistan. Another interesting review in this area was performed by [25], who stated that there were not enough relevant reviews at the European level on oral, nasopharyngeal, and other pharyngeal cancers. The authors obtained data from the Global Burden of Disease 2019 (GBD) on mortality rates, years of life lost, years of life lived with disability, and disability-adjusted life years. These cancers were more common in Eastern Europe, peaking in Hungary. Other authors such as Damgacioglu et al. [26] drew attention to the inconsistency of research on oropharyngeal cancer incidence and mortality rates in the US. The analysis covered the period 2001–2017 and found that the incidence was higher among men than women. The same trend was found for mortality rate by gender. As a result, the authors proposed urgent improvements in prevention and future studies to understand the etiological reasons for geographic disparities related to this cancer.

In the case of HPV-attributable cancers, there has been little analysis globally, as stated by [27]. The authors accessed information from the Cancer Incidence in Five Continents (CI5plus) database, and their analysis covered the 1990–2012 time period. Based on these data and using Bayesian analysis, the authors defined a pre-visional model of HPV-attributable cancer incidence in each country by 2030. The results of the analysis supported the idea of disparity between the countries analysed in relation to this type of cancer. While some countries, such as Uganda, experienced an increase in the incidence rate of this type of cancer, developed countries showed a U-shaped trend. Most countries showed a decreasing or stable trend in the number of cases attributable to HPV. There were exceptions to this trend, such as the US, Japan, the UK, and the Netherlands, which experienced an upward trend over the projection period.

Colorectal cancer, according to Cardoso et al. [28], was a major challenge at the European level. The authors performed an analysis of this type of cancer using indicators of colorectal cancer incidence, mortality, and stage distribution in relation to the implementation of dedicated screening. The analysis covered 21 European countries based on the WHO database. The analysis showed that the incidence of colorectal cancer had decreased substantially over time in countries such as the Czech Republic, Austria, and Germany due to the implementation of long-term screening programmes using colonoscopy and faecal tests. European countries that had not widely implemented screening programmes, such as Bulgaria, Estonia, Norway, and Ukraine, were facing a rising incidence of colorectal cancer. Colorectal cancer incidence and mortality trends were analysed by Wong et al. [29] according to age, sex, and anatomical location in 39 WHO member states. The authors compared colorectal cancer incidence with the human development index in each country analysed, concluding that there were large disparities between continents and groups of countries on the same continent in relation to the indicators analysed. An interesting finding regarding the incidence of early-onset colorectal cancer (CRC) was reported by Akimoto et al. [30], in which the incidence of this type of cancer was particularly increased in high-income countries. The causes were related to diet, obesity, physical inactivity, and antibiotic use. The authors considered it necessary to establish a strategy to improve colorectal cancer prevention and clinical treatment. The same theme was addressed by Xi & Xu [31], who proposed the development of a system to accurately classify CRC subtypes. The authors presented the global epidemiology of CRC in 2020 and forecasted the evolution of this cancer to 2040. A study on colorectal cancer incidence and mortality at the national level in Romania was conducted by Ionescu et al. [32], who used statistical data on all cases registered in hospitals in this country according to the ICD−10 revision (codes C18-C20) during 2016–2018. The results of the data analysis showed that the mortality rate of colorectal cancer in Romania was almost twice the European average, with regional disparities between the north and south of the country. Colorectal cancer health care resources were studied by Henderson et al. [33]. The authors considered primary care, ambulatory care, accident and emergency care, hospital care, and medicines. For the analysis, the authors obtained statistical data from the WHO, OECD, and Eurostat databases and from national entities. On the basis of this data, a picture was drawn of the resources available to fight colorectal cancer and proposals were made to improve the situation at global and national levels.

Other authors examined pancreatic cancer and lung cancer mortality in women [34]. The analysis focused on the five most populous EU member states and the UK, covering the period 1970–2015. The statistics came from the official databases of the World Health Organization and Eurostat. The modelling involved the use of a Joinpoint regression model. The results of the analysis showed that rates for pancreatic cancer in EU men aged 25–49 and 50–64 years had decreased, but they had increased for those aged 65 years and older over the period analysed. The authors drew attention to the need for more effective measures to reduce tobacco use, which accounted for a third of all cancer deaths in the EU. Pancreatic cancer was also analysed by Huang et al. [35] across gender and age groups. Authors such as Khalaf et al. [36] considered pancreatic cancer to be the seventh leading form of cancer worldwide. The analysis covered the period 1975–2016 and used information and data from nine National Cancer Institute registries and the Surveillance, Epidemiology, and End Results Study. The authors quantified the impact of pancreatic cancer by age, gender, race, specific incidence, survival rates, and observed trends. An analysis on the same topic, but over the period 1990 to 2019, was performed by Yu et al. [37]. The authors used data from the Global Burden of Disease (GBD) Study for the EU27 and UK and forecasted the evolution of this cancer to 2039. The modelling was based on Bayesian age-period cohort analysis. The analysis highlighted the trends of increasing incidence and mortality rates of this type of cancer, and the authors highlighted the need for future health policies and interventions. The quality of care of pancreatic cancer patients was studied by Aryannejad et al. [38], with the authors proposing a quality of care index (QCI) resulting from the interpretation of the results of the GBD 2017 study. Japan, the US, and Australia had the highest values of this index, with African countries at the opposite pole. There were also large variations depending on the age of the patients. Authors such as Tonini & Zanni [39] considered pancreatic cancer to have the worst prognosis, as the 5-year survival rate is less than 10%. The causes of this negative prognosis are the late detection of this type of cancer and limited surgical interventions in terms of the number of patients and medical efficacy. The authors conducted an inventory of pancreatic cancer identification and treatment procedures in 2021.

An interesting analysis of gastrointestinal cancers by Xie et al. [40] found that these cancers had the highest number of new cases and deaths each year. There was great disparity in the distribution of these cancers: oesophageal and gastric cancers were strongly present in developing countries, while colorectal cancer was dominant in developed countries in the West. The authors’ analysis of the US, China, and Europe highlighted the link between these cancers and lifestyle habits. The authors retrieved information on pancreatic cancer incidence and mortality in 184 countries from the GLOBOCAN database and modelled it using regression. The results of the analysis spoke of a direct connection between the high incidence and mortality of these types of cancer and smoking, alcohol consumption, physical inactivity, obesity, hypertension, and high cholesterol. The population with the highest exposure to these types of cancer was women aged 50 and older. Another study on gastric cancer by Park et al. [41] focused on the population of Korea and covered the period 1999–2019. The analysis looked at incidence and mortality rates, disease stage, and treatment. It was found that incidence and death rates of this type of cancer significantly decreased between 2011 and 2019, while the proportions of patients with cardiac and fundic cancers remained constant.

Regarding lung cancer, considered the leading cause of cancer-related mortality worldwide, researchers such as Oudkerk et al. [42] discussed new treatment approaches related to the introduction of molecular targets and immunological checkpoint agents. The authors took into account the results of dedicated studies such as the US National Lung Screening Trial (NLST) and NELSON conducted nationwide in the US. The conclusion of the analysis was that the mortality rate for this type of cancer showed a downward trend due to superior screening. This approach was also valid in the UK and supported by acceptable cost effectiveness. The connection between long-term exposure to low-level air pollution and lung cancer incidence was studied by Hvidtfeldt et al. [43] at the European level. The authors modelled the phenomenon based on hybrid models incorporating elements related to air pollution, land use, nitrogen dioxide dispersion, fine particles, black particles, black carbon, and ozone. The next step in the modelling was to use stratified Cox proportional hazard models, resulting in the finding that long-term exposure to ambient fine particles was associated with lung cancer incidence even at concentrations below current EU and WHO limit values. The connection between lung cancer and fine particulate matter was also studied by other specialists, such as Hvidtfeldt, Chen, et al. [44]. The authors studied seven cohorts in Europe for particles of copper, iron, zinc, sulphur, nickel, vanadium, silicon, and potassium. As in the previous study presented above, the authors used stratified Cox proportional hazards models and concluded that there was a positive association between exposure to all eight components and lung cancer incidence. A revolutionary treatment for lung cancer, in the view of Kerr et al. [45], is the use of small molecule inhibitors and the development of immunotherapies. The authors performed a comparative analysis of oncogene driver mutations occurring in the development of this type of cancer in Europe and identified the best strategies in relation to biomarker testing at diagnosis and during treatment. Trends in lung cancer deaths were analysed by Yang et al. [46] globally over the period 1990–2017. The statistical information was obtained from the Global Burden of Disease Survey 2017 and focused on age-standardised mortality rates. The main conclusion of the analysis was that in addition to smoking, the corresponding number of lung cancer deaths had steadily increased due to ageing and population growth.

The high costs associated with treatments for chondral lymphocytic leukaemia were analysed by Yao et al. [47] at global, regional, and national levels over the period 1990–2019. The authors used the database from the Global Burden of Disease study and performed age, gender, and regional structuring of the cancer population. The analysis showed an increase in the number of men and older people affected due to risk factors such as high body mass index and smoking. Using the same database (Global Burden of Disease 2019), other authors such as Ou et al. [48] focused on quantifying the costs of this type of cancer globally. The analysis covered the period 1990–2019 and used the following indicators: incidence, deaths, disability-adjusted life years, and age-standardised corresponding rates by influenza in direct relation to leukaemia. Bayesian modelling enabled the prediction for 2030 that the incidence of leukaemia would increase globally, while the death rate would decrease slightly as risk factors such as smoking, high body mass index, and occupational exposure to benzene or formaldehyde were identified. Investigating risk factors and epidemiological trends in leukaemia, Huang, Chan, Ngai, Lok, Zhang, Lucero-Prisno, et al. [49] focused on indicators such as overall incidence, mortality, associated risk factors, and temporal trends in leukaemia by sex, age, and country. Official statistical information was obtained from GLOBOCAN, CI5, WHO mortality database, NORDCAN, and SEER. Based on regression analysis, the authors highlighted trends and risk factors in the development of cancers globally. Countries such as Germany, Korea, Japan, Canada, and the UK were experiencing a rising trend in the incidence of leukaemia. In other countries, such as the Philippines, Ecuador, Belarus and Thailand, cancer deaths were increasing. A recent study looking at the evolution of leukaemia in the Czech Republic, Hungary, and Poland was re-conducted by Kósa et al. [50]. The authors found that leukaemia patients in these countries who received first-line FCR therapy experienced increased risk of secondary malignancies.

A comprehensive study realised by Sekeroglu & Tuncal [51] analysed lung, breast, colorectal, prostate, and all cancers, which have the highest incidence and mortality rates. The analysis was based on the 2018 Cancer Datasheet of the World Health Organization and covered European countries. Data modelling was performed using linear regression, support vector regression, the decision tree method, a backpropagation neural network, and a radial basis function neural network.

The financial costs of cancer treatment were quantified by Ira-gorri et al. [52]. The authors conducted a meta-analysis in the field to highlight the medical and non-medical costs incurred in cancer treatment by patients and their caregivers. The analysis showed that cancer treatment costs were highest in the US, followed by those in Western Europe, Australia, and Canada. These costs also varied greatly depending on the type of cancer being investigated. As a result, cancer patients and their carers spent between 16% and 42% of their annual income on treatment in low- and middle-income countries. The financial costs incurred by patients diagnosed with cancer were analysed by Fitch et al. [53] based on a Canada-wide questionnaire. Forty percent of these individuals indicated that they had experienced financial hardship and emotional distress during treatment. The authors proposed to the provision of adequate intervention services to patients with precarious financial standing. A solution to decrease the financial burden related to cancer treatment was proposed by Ferraresi et al. [54] with the introduction of centralised procurement within the regional health system in direct reference to regional health systems in Italy. The analysis showed that this procurement process reduced health expenditure per capita by about 2–8% without affecting the level of health-related public services.

A comparative analysis between the WHO List of Essential Medicines for Cancer and the Oncologists’ Priority Medicines List was conducted by Fundytus et al. [55] based on an international cross-sectional survey developed and disseminated to a global network of oncologists in 89 countries and regions. The survey questionnaire asked oncologists to select ten cancer drugs that provide the greatest public health benefit for each state. The most commonly selected drugs were doxorubicin, cisplatin, paclitaxel, pembrolizumab, trastuzumab, carboplatin, and 5-fluorouracil. Of the medicines selected by respondents, only 60% were on the WHO Essential Medicines List for cancer. Regarding cancer drugs, Godman et al. [56] conducted an analysis on the pricing of new cancer drugs. The authors considered potential pricing approaches based on minimum effectiveness. In the context of the development of a large number of drugs to treat cancer, the authors used multi-criteria decision analyses to weigh the tendency for continued high prices against the magnitude of confidential reductions for reimbursement.

Across Europe, the incidence of cervical cancer varies widely. To eliminate this type of cancer, European countries should use the human papillomavirus (HPV) vaccine in at least 90% of girls up to the age of 15 by 2030. As a result, authors such as Arbyn et al. [57] proposed that the European Health Authorities develop the third edition of the “European Guidelines for Quality Assurance in Cervical Screening”, for vaccine-based cervical cancer prevention and integrated HPV screening, thus monitoring progress towards the elimination target.

People with cancer and their families need to be proactive during diagnosis and treatment of the disease, as stated by Howell et al. [58]. In this regard, the authors highlighted six priority action areas covering actions starting with preparing patients and survivors for active engagement in care (Action 1) and ending with expanding outreach and access to support programmes (Action 6).

Geographical disparities in the incidence and mortality due to hepatocellular carcinoma in the UK were studied by Burton et al. [59] over the period 2010–2016. The analysis considered indicators such as age-standardised incidence rates and net survival adjusted for age at diagnosis, sex, and geographical area. Incidence rates for this type of cancer increased for both men and women and were influenced by regional levels of socio-economic development, quality of early diagnosis, and curative treatment.

Incidence and mortality rates related to malignant peritoneal mesothelioma during 2000–2018 were studied by Calthorpe et al. [60] according to age, sex, and histology. For the estimation of incidence rates, the authors used Joinpoint regression and for mortality rates, they used multivariable Cox proportional hazards models. The results of the analysis supported the idea that while the incidence of this type of cancer has remained relatively constant over time, mortality rates have decreased due to new surgical intervention procedures.

The impact of COVID−19 on the treatment of cancer patients worldwide was analysed by [61] at the population level in Canada using a microsimulation model. The model took into account variables such as cancer incidence, stage at diagnosis, and mortality. Using 10 stochastic simulations, the authors concluded that interruptions in cancer care during the COVID−19 pandemic may lead to a 2% increase in cancer deaths in Canada by 2030. The highest mortality rates were for breast, lung, and colorectal cancers.

Correlations between the level of economic development and cancer incidence and mortality were highlighted by Mao et al. [62], who focused on low- and middle-income states. The authors quantified statistical data on cancer prevention, treatment, palliative care, and survival. A first observation was that the approach to cancer in these states was predominantly through traditional, complementary, and integrative methods, which were cheaper. As a compromise solution, it was proposed to combine conventional and traditional medicine methods in the treatment of different cancers.

An interesting approach is to understand the connection between a cancer’s detection and treatment and its incidence and mortality rates. This connection varies from one cancer to another. In the case of oral cancer, Ribeiro et al. [63] found that “screening by visual inspection is capable of identifying lesions at early stages, increasing survival and decreasing oral cancer incidence and mortality.” The authors drew on official databases such as MEDLINE/PubMed, Cochrane Library, EMBASE, and LILACS, covering people aged 18 to 60 years. The main conclusion of the study was that implementing effective screening can reduce oral cancer incidence and mortality rates. An important element in decreasing the incidence of oral cancer is the level of awareness of those at risk. A study on this subject in India was performed by Singla et al. [64], covering rural areas and trying to explain the causes of the high incidence of oral cancer in this country using a dedicated questionnaire. The results of the questionnaire pointed to the lack of an effective network of screening centres, smoking addiction, and lack of adequate information on the subject as the main causes of the increased incidence of oral cancer. Moreover, 90% of the people surveyed had undergone some form of alternative treatment before consulting a cancer specialist. Eliminating these disruptive factors can help to reduce oral cancer incidence and mortality rates. A large study conducted at the US level over the period 2012–2019 by Tranby et al. [65] used statistical data from databases such as Medicaid and IBM Watson Health MarketScan. The authors applied regression analysis to process the data and found that incidence and mortality trended downward. On the other hand, the incidence was higher among older, male, white adults who used tobacco or alcohol or had human immunodeficiency virus/acquired immunodeficiency syndrome. The incidence rate was lower in people who had regular dental check-ups. The authors stated that lowering the incidence rate in a developed country like the US also has the effect of lowering the mortality rate. The incidence, mortality, and mortality–incidence ratio related to oral cancer were analysed by L. Xie & Shang [66] in connection with the human development index. The analysis covered China over the period 1990–2019 and used statistical data from Global Burden of Disease 2019. Using correlation analysis and regression analysis, incidence rates by age group, mortality rates by age group, and different stages of the human development index were evaluated. The main conclusion of the analysis was that the public authority should continue to allocate additional funds for the prevention and treatment of human cancer. This is only possible under conditions of long-term economic growth. Another study on oral cancer incidence and mortality was conducted by Zhu et al. [67] based on the APC model used for the analysis of age characteristics. The mortality rate of oral cancer showed a decreasing trend, while the rate related to tobacco chewing showed a significantly increasing trend. The oral cancer mortality rate was higher in men, and the age of death attributed to each risk factor for this cancer varied between 45 and 74 years. The incidence and mortality for oral cancer caused by alcohol and smokeless tobacco increased in age groups younger than 45 years. In regions with traditionally high incidence, such as India and Pakistan, oral cancer incidence rates showed a downward trend.

In the case of colorectal cancer, Zorzi & Urso [68] argued that faecal immunochemical tests were the most effective screening tools for colorectal cancer globally, but this was complemented by the idea that there were as yet no randomised controlled trials that showed the link between these procedures and incidence and mortality rates for this type of cancer. The authors started from the Italian experience in the field but noted that the use of faecal immunochemical tests represents an unplanned experimental design. Nevertheless, these tests had a positive impact on reducing incidence and mortality rates and improving the endoscopic treatment rate of early invasive lesions. It was noted that faecal immunochemical tests were less efficient for the proximal colon than for the distal colon and rectum. Colorectal cancer is the third and second most commonly diagnosed cancer in men and women, respectively. In their opinion, Gurba et al. [69] stated that standard antitumor therapies, including cisplatin, were no longer effective and created drug resistance and severe side effects. New colorectal cancer treatments include platinum-, gold-, silver-, iridium-, or ruthenium-based drugs. In the case of gold-based drugs, they show good solvency in water but they are not stable under physiological conditions. Research is focused on stabilisation of the Au(III) cation. In order to decrease the incidence rate of colorectal cancer, Cervantes et al. [70] proposed radiological imaging and appropriate histology of the primary tumour or metastases before any treatment was given as an effective solution. The authors studied tissue handling procedures for biomarker testing in 10% neutral-buffered formalin solution (4% formaldehyde). Before starting first-line treatment, RAS testing was recommended. Second and third line treatments were recommended to be initiated with cetuximabencorafenib. This approach may also lead to reduced mortality rates in colorectal cancer. According to K.-X. Yu et al. [71], socioeconomic deprivation has a major impact on colorectal cancer incidence and mortality. Using a sample of patients diagnosed with colorectal cancer during 2007–2015, the authors conducted a survival study using the Area Deprivation Index (ADI). The statistical data were modelled using specific methods (inverse probability weighting method and multiple regression) and revealed that patients with high ADI had worse presenting characteristics and lower colorectal cancer survival after treatment than their counterparts with low ADI. The Dutch experience in treating colorectal cancer was highlighted by Smits et al. [72] based on the study of histological slides of adenomas with high-grade dysplasia and early colorectal carcinomas from 20 laboratories. The results of the analysis highlighted inter-observer variations in histopathological examination with effects on treatment options. Reducing these variations may mitigate colorectal cancer incidence and mortality rates.

In pancreatic cancer, Andersson et al. [73] noted that there have been no major advances in early diagnosis and new targeted therapies. The “classical” treatment methods (surgery and chemotherapy) also have limitations. The authors proposed the development of molecular methods in the investigation and treatment of this type of cancer and performed a meta-analysis of treatment methods in relation to incidence and mortality rates for this cancer. Starting from the impact of pancreatic cancer (the seventh leading cause of cancer deaths worldwide), Ettrich et al. [74] drew attention to the increasing incidence rates for this type of cancer and noted that there was currently no treatment other than surgical resection in combination with adjuvant systemic chemotherapy. Even though the mortality rate has fallen to 54%, this treatment often leads to micrometastases. An improvement in the approach to pancreatic cancer is targeted therapies for the individualisation of treatment (BRCA1/2 or BRCA1). Using the MEDLINE database, Coll-Ortega et al. [75] performed a meta-analysis in the field over the period 2000–2018 (33 studies) in relation to the centralisation of pancreatic cancer surgeries using models related to designated hospitals, defining minimum volumes per provider, and/or recommendations included in national protocols and guidelines. The authors advocated surgical management of this cancer by multidisciplinary specialist teams and implementation of hospital management in line with the recommendations proposed in the EU Bratislava initiative through the Declaration for Pancreatic Cancer Care. Centralising pancreatic cancer care within an integrated regional health care system was the subject of research by Hsu et al. [76], who found that this approach led to lower mortality rates. The questionnaire-based statistical analysis covered the period 2010–2018 and four centres of excellence (COEs) in surgery, medical oncology, and other specialties. A first conclusion of the analysis was that neoadjuvant chemotherapy contributed to a reduction in mortality rates. The authors believed that an integrated system of care would improve early detection rates of pancreatic cancer and reduce mortality through the use of neoadjuvant therapy. In the case of the US, pancreatic cancer has been characterised by steadily increasing incidence and mortality rates, as stated by Rosenzweig et al. [77]. The authors first reviewed national treatment guidelines in the field (Scientific Action Network (PanCAN) of Pancreatic Cancer Action Network (PanCAN) and Medical Affairs) and focused on diagnosis, treatment, and care for pancreatic cancer.

The impact of CT screening on lung cancer incidence rates, mortality, and the percentage of early stage diagnosis was quantified by Emmerick et al. [78] through a retrospective analysis of the SEER−18 time series over the period 2006–2016. The analysis considered indicators such as age, gender, race, marital status, insurance status, and household income. Lung cancer incidence and mortality rates were found to have decreased over the period analysed. The authors believed that improving diagnosis of early-stage lung cancer through comprehensive screening and reducing disparities in access to diagnosis could reduce mortality rates. An analysis of lung cancer incidence and mortality in 185 states was performed by Zhou et al. [79]. The authors drew on GLOBOCAN, Cancer Incidence in Five Continents, and World Health Organization databases. The analysis of incidence and mortality rates for this type of cancer showed a decrease for men in most countries, but an increase for women. Incidence rates had decreased even more for young people. Current lung cancer treatment covering drugs with targeted monoclonal antibodies or chimeric antigen receptor-modified (CAR-T) antibodies, however, have limitations in their design, as described by Xu et al. [80]. These innovative treatments have to cope with challenges related to tumor lineage, neurotoxicity, and cytokine release syndromes. A comprehensive review conducted over the period 2015–2020 by Frost et al. [81] focused on the clinical characterisation, diagnosis, risk factors, and treatment of lung cancer. The analysis covered high-volume lung cancer centers in Berlin and was based on a questionnaire completed by patients who received checkpoint inhibitors (CPI). It found that chest radiotherapy targeting the lung was the only independent risk factor for lung cancer. Authors such as Xiao et al. [82] discussed procedures such as single-use low-dose spiral computed tomography (LDCT) for improving early-stage diagnosis and reducing lung cancer mortality. The analysis covered the period 2021–2018 and was based on a competing risk model used to develop the nomogram. In evaluating the nomogram, it was believed that more effective disease management procedures could be capable of reducing lung cancer mortality rates.

Leukaemia is a difficult cancer to treat, in the opinion Howlader et al. [83], who recommended molecular targeted therapies such as tyrosine kinase inhibitors (TKIs). The authors assumed that mortality trends reflect the combined effects of incidence and survival in leukaemia. The analysis covered the period 1992–2017 and US adults by subtype and year of diagnosis. The results of the analysis showed that incidence rates fluctuated over the period analysed, while mortality rates decreased with the use of TKIs. The multiplicity of leukaemia manifestations makes it necessary to implement new treatment strategies and develop new therapies, in the opinion of Chhikara & Parang [84]. The authors noted that the newly developed drugs and diagnostic methods help to only partially cure leukaemia. A comparative statistical analysis of the current prevalence of leukaemia in line with advances in theranostics was recommended. Leukaemia and the four main subtypes were the subject of an analysis conducted by Du et al. [85] in 204 states over the period 1990–2019. The authors also forecasted the evolution of the incidence and mortality rates up to the year 2030, showing a decrease in incidence and mortality rates globally over the period analysed. Both rates were higher for men than women. Smoking was the main cause of death in men, while high body mass index was the main cause of death in women. Leukaemia cases in France diagnosed between 2012–2016 were analysed by Atsou et al. [86] according to age, medical unit of entry, and access to specialised haematology services. Statistical regression analysis highlighted that a positive impact on leukaemia mortality rates was due to access to cytogenetic testing and curative treatment tests in turn, as well as early treatment. A comprehensive study covering 204 countries over the period 1990–2019 was performed by Xiang et al. [87]. The authors used gender, age, sociodemographics (SDI), region, etc. as indicators to assess the trends in incidence and mortality rates by this type of cancer. Analysis of the dedicated statistical data revealed a slow increase in the incidence and a slight decrease in the mortality of leukaemia.

Authors such as Yansheng Yao et al. [88] believed that brain cancer has increasing incidence and mortality rates year after year. Treatment of this type of cancer consists of surgery followed by radiotherapy and chemotherapy, but the side effects are multiple. As a result, a new approach to the treatment of brain cancer is needed. One solution may be nanodrugs based on ferroptosis. In addition, the authors conducted an inventory of solutions for the development of ferroptosis-based nanodrugs against brain tumors. This development could consistently decrease the mortality rate of this type of cancer. An analysis of brain cancer in the US by histopathological grouping, age, race, and sex was performed by Thierheimer et al. [89] for the period 2004–2018. Using modelling, incidence and mortality rate trends were defined, providing valuable solutions for public health planning. The reduction of brain cancer mortality was the subject of research by Cioffi et al. [90] based on a comparative analysis covering the periods 2004–2007, 2008–2012, and 2013–2017. Mathematical modelling restricted by sex, race/ethnicity, and treatment showed that mortality rates remained relatively constant in patients aged 40–64 years, while they decreased in younger patients. Joinpoint analysis was used by Che et al. [91] to highlight changes in brain cancer incidence and mortality over the past 10 years. The results of the analysis showed a decrease in incidence and mortality rates. An interesting approach linking brain cancer to cognitive–emotional–behavioral therapy for patients was presented by Chieffo et al. [92]. The authors considered that at the time of brain cancer diagnosis, patients exhibit cognitive–emotional–behavioral traits that are detectable.

Based on the study of the literature, researchers’ concerns have emerged about how to diagnose and treat these cancers in order to limit increases in incidence and mortality rates, reflecting new trends in the literature. The main guidelines support the implementation of high-quality screening in the early diagnosis of cancer, the extension of screening procedures to the entire population at risk of disease, the strengthening of risk factor control (smoking, obesity, physical inactivity, alcohol consumption, and infections), the development of public–private partnerships in the field, the development of new surgical intervention procedures, the funding of holistic approaches to cancer control and management, as well as the combination of conventional and traditional medicine methods in the treatment of different cancers.

## 3. Materials and Methods

In order to carry out this scientific work, we accessed official statistical information from the WHO, World Bank, and Eurostat [1,93,94,95,96,97]. Based on this information, we built a dedicated database with the following indicators (see Table 1):

The study covers the period 1993–2021 and 25 EU member states, as there is no official reporting for Luxembourg and Cyprus.

The database was subjected to analysis and consolidation processes requiring procedures to concatenate multiple databases and monitor data comparability.

After building the consolidated database, the following working hypotheses were designed:

**H1.**  *There is a strong indirect connection between the level of economic development of states and mortality rates of lip, oral cavity, and pharynx, lung colon, brain and central nervous system cancers, with results being differentiated by sex. This hypothesis is also supported by the results of research carried out by Belot & Pohar-Perme; Dyba et al.; Ferraresi et al.; Fitch et al.; Hvidtfeldt, Chen, et al.; Hvidtfeldt, Severi, et al.; Iragorri et al.; Kerr et al.; Oudkerk et al.; and Yang et al. [22,23,42,43,44,45,46,52,53,54].*

**H2.**  *There is a weak direct connection with the decreasing trend in intensity between the level of economic development and leukaemia and pancreatic cancer mortality rates, with results differentiated by gender. The results of research realised by Aryannejad et al.; Carioli et al.; Huang et al.; Huang, Chan, Ngai, Lok, Zhang, Lucero-Prisno, et al.; Khalaf et al.; Kósa et al.; Ou et al.; Tonini & Zanni; Yao et al.; and Yu et al. [34,35,36,37,38,39,47,48,49,50] support our hypothesis.*

**H3.**  *There is a strong indirect bidirectional connection for men and a strong direct bidirectional connection for women between the level of economic development of countries and mortality rates of lung and lip, oral cavity, and pharyngeal cancers. The hypothesis is supported by research results from Duran-Romero et al.; Hvidtfeldt, Chen, et al.; Hvidtfeldt, Severi, et al.; Kerr et al.; O’Sullivan et al.; Oudkerk et al.; Sofi-Mahmudi et al.; and Yang et al. [24,25,42,43,44,45,46,98].*

**H4.**  *There is a weak direct connection with the decreasing trend in intensity between the level of economic development and incidence rates of leukaemia, colon, pancreatic, and brain and central nervous system cancers, with results being differentiated by gender. This hypothesis is reflected in the results of research by [2,3,4,5,6,7,8,9,10].*

Based on the research hypotheses, we defined the following research model:

(1) $$\begin{array}{cc} PRCPPP\left(year\right) & =\alpha \cdot COFOG\left(year\right)+\beta \cdot IncidenceLip(year)+\gamma \\ & \cdot MortalityLip(year)+\delta \cdot IncidenceColon(year)+\theta \\ & \cdot MortalityColon(year)+\vartheta \cdot MortalityPancreas(year)+\mu \\ & \cdot IncidencePancreas(year)+\pi \cdot IncidenceLung(year)+\rho \\ & \cdot MortalityLung(year),+\sigma \cdot MortalityLeukaemia(year)+\tau \\ & \cdot IncidenceLeukaemia(year),+\varphi \cdot MortalityBrain(year)+\omega \\ & \cdot IncidenceBrain(year)+\varepsilon \end{array}$$

where: PRCPP—purchasing power parities (PPPs), price level indices, and real expenditures (dependent variable); COFOG—general government expenditure by function; IncidenceLip—incidence of lip, oral cavity, and pharyngeal cancer; MortalityLip—mortality of lip, oral cavity, and pharyngeal cancer; IncidenceColon—incidence of colon cancer; MortalityColon—mortality of colon cancer; IncidencePancreas—incidence of pancreatic cancer; MortalityPancreas—mortality of pancreatic cancer; IncidenceLung—incidence of lung cancer; MortalityLung—mortality of lung cancer; IncidenceLeukaemia—incidence of leukaemia; MortalityLeukaemia—mortality of leukaemia; IncidenceBrain—incidence of brain and central nervous system cancer; MortalityBrain—mortality of brain and central nervous system cancer (regresori); α, β, γ, δ, θ, ϑ, μ, π, ρ,σ,τ,φ,ω—coefficients of regression variables; and ε—residual value.

On the basis of the general model, using IBM-SPSS25 software, the regression equations were dynamically projected over the period 1993–2021 and are presented (in terms of coefficients) in histogram form in Table 2.

From the model equation presented, it appeared that the values of the correlation coefficients varied and were inversely proportional (red colour) to the dependent variable (PRCPPP) but were also directly proportional (blue colour), which led us to conclude that we cannot speak to unitary public policies in the field of prevention among the increasingly economically independent population.

The dynamic analysis of the variation of the regression coefficients allowed us to observe the differentiated evolution of the correlation between the standard of living and the incidence and mortality rates of different types of cancers, as follows:

- As far as lip, oral cavity and pharyngeal cancer was concerned, the increase in its incidence for men varied between 3.08 units per 100,000 inhabitants, while the economic wealth of men increased by one unit in 1993. In 2021, the incidence rate became 0.381 for an increase of one unit in the economic well-being of the men in the sample analysed. The maximum value was reached in 1994, when the incidence rate was 3.82 units for one unit increase in men’s economic well-being, while the minimum value was recorded in 2012, with a negative incidence rate with a correlation coefficient of −1.72 related to the dependent variable increase in men’s economic welfare.
- Analysis of the disease situation for the female population in the 25 EU member states for the lip, oral cavity, and pharyngeal cancer type showed a much wider variation in the indicator, from 4.06 incidence points in 1993 to −0.68 points in 2021. The maximum value was reached in 1996, when the incidence rate was 22.65 units for one unit increase in women’s economic well-being, while the minimum value was recorded in 1998, i.e., a negative incidence rate with a correlation coefficient of −7.17 related to the dependent variable increase in women’s economic welfare.
- As far as lip, oral cavity, and pharyngeal cancer was concerned, the increase in its mortality for men ranged from −8.77 units per 100,000 inhabitants to −8.77 units per 100,000 inhabitants, while men’s economic well-being increased by one unit in 1993 (maximum value). In 2021, the mortality rate became −1.3 for an increase of one unit in the economic well-being of men in the analysed sample (minimum value).
- Analysis of disease mortality for the female population in the 25 EU member states for the lip, oral cavity, and pharyngeal cancer type showed a much wider variation in the indicator, from −35.3 incidence points in 1993 to −6.7 points in 2021. The maximum value was reached in 1994, when the mortality rate was −51.17 units for one unit increase in women’s economic well-being, while the minimum value was recorded in 2019, i.e., negative mortality rate with a correlation coefficient of −4.55 related to the dependent variable increase in women’s economic welfare.
- As far as colon cancer was concerned, the increase in its incidence varied for men between 2.22 units per 100,000 inhabitants, while the economic wealth of men increased by one unit in 1993. In 2021, the incidence rate became 1.03 for an increase of one unit in the economic welfare of the men in the sample analysed. The maximum value was reached in 1996, when the incidence rate was 5.7 units for one unit increase in men’s economic well-being, while the minimum value was recorded in 2001, namely a negative incidence rate with a correlation coefficient of −1.3 related to thedependent variable increase in men’s economic welfare.
- Analysis of the disease situation for the female population in the 25 EU member states for colon cancer showed much wider variation, from 3.11 incidence points in 1993 to 1.21 points in 2021. The maximum value was reached in 1995, when the incidence rate was 4.61 units for one unit increase in women’s economic well-being, while the minimum value was recorded in 2010, i.e., a negative incidence rate with a correlation coefficient of −2.08 related to thedependent variable increase in women’s economic welfare.
- As far as colon cancer was concerned, the increase in colon cancer mortality for men ranged from −5.9 units per 100,000 inhabitants, while the economic welfare of men increased by one unit in 1993 (maximum value). In 2021, the mortality rate became −2.95 for an increase of one unit in the economic welfare of the men in the sample analysed (minimum value). The maximum value was reached in 2012 (1.66) and the minimum was reached in 1994 (−9.12).
- Analysis of disease mortality for the female population in the 25 EU member states for the colon cancer type showed a much wider variation of the indicator, from −2.77 incidence points in 1993 to −4.98 points in 2021. The maximum value was reached in 2009, when the mortality rate was 2.02 units for one unit increase in women’s economic welfare, while the minimum value was recorded in 1995, i.e., a negative mortality rate with a correlation coefficient of −7.07 related to the dependent variable increase in women’s economic welfare.
- As far as pancreatic cancer was concerned, the increase in its incidence in men ranged from −9.9 units per 100,000 inhabitants, while the economic welfare of men increased by one unit in 1993. In 2021, the incidence rate became −1.93 for an increase of one unit in the economic welfare of the men in the sample analysed. The maximum value was reached in 2014, when the incidence rate was 4.35 units for the one unit increase in men’s economic welfare, while the minimum value was recorded in 1998, with a negative incidence rate with a correlation coefficient of −13.62 related to the dependent variable increase in men’s economic welfare.
- Analysis of the disease situation for the female population in the 25 EU member states for pancreatic cancer showed a much wider variation in the indicator, from −8.99 incidence points in 1993 to −2.69 points in 2021. The maximum value was reached in 2013, when the incidence rate was 1.49 units for one unit increase in women’s economic welfare, while the minimum value was recorded in 1998, i.e., a negative incidence rate with a correlation coefficient of −11.11 related to the dependent variable increase in women’s economic welfare.
- As far as pancreatic cancer was concerned, the increase in pancreatic cancer mortality for men ranged from 21.87 units per 100,000 population, while the economic welfare of men increased by one unit in 1993. In 2021, the mortality rate became 0.06 for an increase of one unit in the economic welfare of the men in the analysed sample. The maximum value was reached in 1994 (23.79) and the minimum value was reached in 2014 (−2.39).
- Analysis of disease mortality for the female population in the 25 EU member states for pancreatic cancer showed a much wider variation in the indicator, from 16.7 incidence points in 1993 to 2.0 points in 2021. The maximum value was reached in 1999, when the mortality rate was 20.5 units for one unit increase in women’s economic welfare, while the minimum value was recorded in 2013, i.e., a negative mortality rate with a correlation coefficient of −0.78 related to the dependent variable increase in women’s economic welfare.
- As far as lung cancer was concerned, the increase in its incidence varied for men between −0.35 units per 100,000 inhabitants, while the economic welfare of men increased by one unit in 1993. In 2021, the incidence rate became 0.27 for an increase of one unit in the economic welfare of the men in the analysed sample. The maximum value was reached in 2011, when the incidence rate was 1.9 units for one unit increase in men’s economic welfare, while the minimum value was recorded in 1998, with a negative incidence rate with a correlation coefficient of −1.6 related to the dependent variable increase in men’s economic welfare.
- Analysis of the disease situation for the female population in the 25 EU member states for the lung cancer type showed a much wider variation in the indicator, from −0.9 incidence points in 1993 to 1.08 points in 2021. The maximum value was reached in 2002, when the incidence rate was 3.78 units for one unit increase in women’s economic welfare, while the minimum value was recorded in 1995, i.e., a negative incidence rate with a correlation coefficient of −4.98 related to the dependent variable increase in women’s economic welfare.
- As far as lung cancer was concerned, the increase in mortality for men ranged from 0.8 units per 100,000 inhabitants, while the economic welfare of men increased by one unit in 1993. In 2021, the mortality rate became −0.8 for an increase of one unit in the economic welfare of the men in the analysed sample. The maximum value was reached in 1998 (1.9) and the minimum value was reached in 2011 (−3.39).
- Analysis of disease mortality for the female population in the 25 EU member states for the lung cancer type showed a much wider variation in the indicator, from 0.42 incidence points in 1993 to −0.63 points in 2021. The maximum value was reached in 1996, when the mortality rate was 4.2 units for one unit increase in women’s economic welfare, while the minimum value was recorded in 2002, i.e., a negative mortality rate with a correlation coefficient of −3.5 related to the dependent variable increase in women’s economic welfare.
- As far as leukaemia was concerned, the increase in its incidence in men varied between 12.4 units per 100,000 inhabitants, while the economic welfare of men increased by one unit in 1993. In 2021, the incidence rate became −0.07 for an increase of one unit in the economic welfare of men in the analysed sample. The maximum value was reached in 2001, when the incidence rate was 12.95 units for one unit increase in men’s economic welfare, while the minimum value was recorded in 2007, with a negative incidence rate with a correlation coefficient of −2.5 related to the dependent variable increase in men’s economic welfare.
- Analysis of the disease situation for the female population in the 25 EU member states for the leukaemia cancer type showed a much wider variation in the indicator, from 6.59 incidence points in 1993 to 1.87 points in 2021. The maximum value was reached in 1997, when the incidence rate was 13.5 units for one unit increase in women’s economic welfare, while the minimum value was recorded in 2005, i.e., a negative incidence rate with a correlation coefficient of −4.07 related to the dependent variable increase in women’s economic welfare.
- As far as leukaemia was concerned, the increase in its mortality for men ranged from −10 units per 100,000 inhabitants while the economic wealth of men increased by one unit in 1993. In 2021, the mortality rate became 2.97 for an increase of one unit in the economic welfare of the men in the analysed sample. The maximum value was reached in 2018 (7.66) and the minimum value was reached in 1997 (−20.5).
- Analysis of disease mortality for the female population in the 25 EU member states for the leukaemia cancer type showed a much wider variation in the indicator, from −5.8 incidence points in 1993 to 2.33 points in 2021. The maximum value was reached in 2008, when the mortality rate was 7.47 units for one unit increase in women’s economic welfare, while the minimum value was recorded in the year 1997, i.e., a negative mortality rate with a correlation coefficient of −21.7 related to the dependent variable increase in women’s economic welfare.
- For brain and central nervous system cancer, the increase in its incidence varied for men between 4.28 units per 100,000 inhabitants, while the economic welfare of men increased by one unit in 1993. In 2021, the incidence rate became 1.19 for an increase of one unit in the economic welfare of the men in the analysed sample. The maximum value was reached in 1997, when the incidence rate was 9.53 units for one unit increase in men’s economic welfare, while the minimum value was recorded in 2016, with a negative incidence rate with a correlation coefficient of −0.57 related to the dependent variable increase in men’s economic welfare.
- Analysis of the disease situation for the female population in the 25 EU member states for the brain and central nervous system cancer type showed a much wider variation in the indicator, from 3.96 incidence points in 1993 to 0.72 points in 2021. The maximum value was reached in 1997, when the incidence rate was 5.1 units for one unit increase in women’s economic welfare, while the minimum value was recorded in 2014, i.e., a negative incidence rate with a correlation coefficient of 0.27 related to the dependent variable increase in women’s economic welfare.
- As far as brain and central nervous system cancer was concerned, the increase in its mortality for men ranged from −15.5 units per 100,000 inhabitants, while the economic welfare of men increased by one unit in 1993. In 2021, the mortality rate became −6.25 for a one unit increase in the economic welfare of men in the analysed sample. The maximum value was reached in 2004 (1.17) and the minimum value was reached in 2001 (−19.12).
- Analysis of disease mortality for the female population in the 25 EU member states for the brain and central nervous system cancer type showed a much wider variation in the indicator, from −13.54 incidence points in 1993 to −10.13 points in 2021. The maximum value was reached in 2001, when the mortality rate was −23.87 units for one unit increase in women’s economic welfare, while the minimum value was recorded in 1997, i.e., a negative mortality rate with a correlation coefficient of −3.4 related to the dependent variable increase in women’s economic welfare.

**Figure 5:**
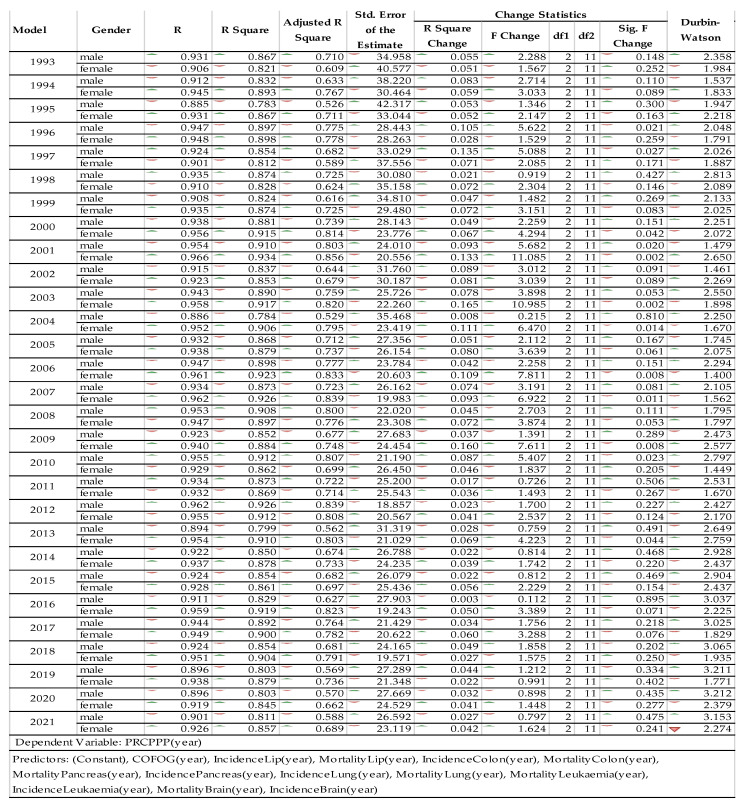
Summary of the dynamic regional cancer model. Realised by authors using IBM-SPSS25 software.

Based on the equations of the regional dynamic cancer model, significance tests were performed for the 29 specific two-branch models (by sex), resulting in the following model summary (see Figure 5).

From the analysis of the multiple regression models, it was shown that the value of the coefficient of determination varied on average between 78.3% and 93.4%, which demonstrated that the models were representative of the studied phenomenon. The value of the F function ranged from 0.112 to 11.25, which meant that there was large variability in individual model values over time, which was also apparent from the analysis of the Durbin–Watson coefficients.

## 4. Results

The results of the application of the general model via ANOVA test in terms of model validation and testing are presented in Table 3.

The ANOVA test analysis showed that the sum of squares regressions were represented in the model in a variable proportion between the minimum point of 81% and the maximum point of 93%. The values of the Sig. coefficient of the F function were between 0 and 0.019, which allowed the validation of the alternative hypothesis and the rejection of the null hypothesis in 19 out of 29 cases for the male sex-differentiated model and in 26 out of 29 cases for the female sex-differentiated model.

Tests of between-model variation showed that the regression models were more favourable to the male population in 1993, 1997, 1998, 2008, 2010, 2011, and 2012. The same analysis showed that 1994, 1995, 1996, 1999, 2000, 2001, 2002, 2003, 2004, 2005, 2006, 2007, 2009, 2013, 2014, 2015, 2016, 2017, 2018, 2019, 2020, and 2021 represented the peak years for the female population to fight cancer.

The analysis (Table 3) showed that since 2013 the results of economic growth have had a predominant impact on improving the clinical situation both in terms of the incidence and mortality for women, except for lip, oral cavity, and pharyngeal cancer. The male population did not have the same response rates to economic stimuli and health expenditure financing as the female population.

The analysis of the correlations between economic welfare growth and the incidence and mortality of various cancers by sex was better highlighted by the Pearson correlation coefficient analysis of the model (see Table 4).

Analysis of the Pearson coefficients showed that for lip, oral cavity, and pharyngeal cancer in the male population, the coefficients varied between −0.169 points and 0.07 points, showing a dilution of the effect of increasing welfare on the incidence rate of this cancer in the last years (2020–2021), which was related to the pandemic. As far as mortality was concerned, significant progress had been made, including in the context of increasing economic welfare, although the amount of care allocated per male patient did not significantly influence the mortality rate in the years of the pandemic crisis. Despite a reduction in financial allocations as a result of the measures taken by the authorities to combat the spread of coronary heart disease, mortality rates among men continued to show a favourable trend towards limiting mortality caused by this disease. Among women, the incidence situation was unfavourable. According to the data presented in Table 4, the higher the economic welfare, the higher the incidence of lip, oral cavity, and pharyngeal cancer. The incidence value of lip, oral cavity, and pharyngeal cancer among the female population increased with economic welfare, showing the highest Pearson correlation values in the years 2014–2019 over the whole range analysed. The lowest correlation values were recorded between 1997 and 2000.

Our study highlighted the importance of monitoring the health status of the population at risk of cancer in relation to social welfare status and by gender. Another study using a similar regression method showed the correlation between health service planning, resource management, and prediction of cancer incidence and mortality [13]. In contrast, our proposed model is more complex, includes more variables, and covers a longer period of time.

## 5. Discussion

**Figure 6:**
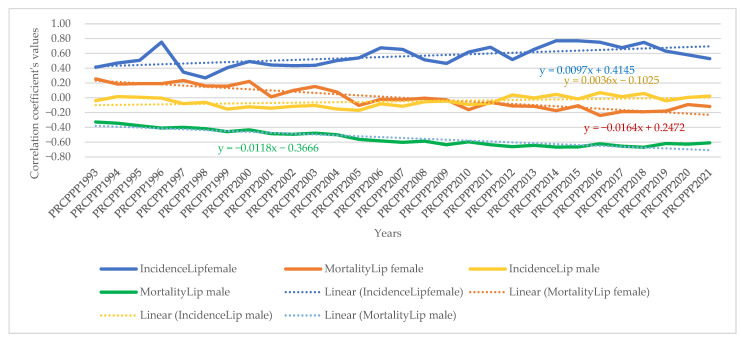
Trends in sex-standardised correlation coefficients between index and mortality rates for lip, oral cavity, and pharyngeal cancer in relation to the dependent variable purchasing power parities (PPPs), price level indices, and real expenditures.

It has been observed in the literature that the decrease in the incidence and mortality rates of lip, oral cavity, and pharyngeal cancer were due to a reduction in tobacco/alcohol consumption in conjunction with careful monitoring of this cancer type by age group, sex, and location as well as continued preventive treatment against known risk factors [24]. According to our research, in terms of mortality due to lip, oral cavity, and pharyngeal cancer, there was an improvement in the sense of a gradual reduction in mortality as the level of economic welfare increased, considering the influence of financial allocations for treatment programmes for this type of cancer. All of these observations and developments confirmed the H1 and H3 working hypotheses regarding lip, oral cavity, and pharyngeal cancer. The above developments are highlighted in Figure 6.

**Figure 7:**
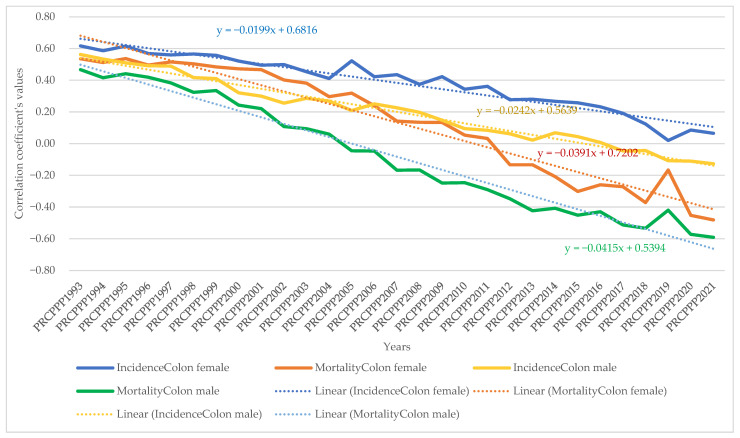
Trends in sex-standardised correlation coefficients between index and mortality rates for colon cancer in relation to the dependent variable purchasing power parities (PPPs), price level indices, and real expenditures.

In the literature, Hossain et al. [19] showed high incidence and death rates for colorectal cancer in developed countries, but there was a significant increase in developing countries. According to the proposed model and the statistical analysis in the dynamics presented above, colon cancer has experienced a favourable evolution in the sense of reduced incidence with increasing economic welfare. In the case of men, the maximum point of the unfavourable directly proportional correlation was recorded in the period 1993–1995, while the minimum point of the favourable inversely proportional correlation was reached in the period 2019–2021. From a mortality point of view, we can say that the economic welfare situation had a much stronger influence on mortality rates in terms of decreasing the mortality rates for the male population. Thus, if the maximum unfavourable values were recorded in the period 1993–1996 when the direct correlation was over 40%, if economic welfare increased by one unit, the mortality rate increased by 0.4 units. The minimum favourable negative values were related to the period 2017–2021 when the levels of favourably negative and inversely proportional correlations were over 505; thus when economic welfare increased by one unit, the mortality rate decreased by 0.5 units. In the case of women, the results of decreasing incidence rates were not as favourable as in the case of men, with only positive and directly proportional correlation values between female colon cancer incidence rates and the level of economic welfare in the 25 countries analysed. However, the values were decreasing, with the maximum level of unfavourable direct correlation being reached in the period 1993–2000 with a correlation of over 50%, while in the period 2019–2021, direct correlations were present with a minimum favourable value of less than 8%. These results meant that in this period the influence of economic welfare on the incidence of colon cancer reached neutrality in relation to the strong unfavourable influence at the beginning of the period analysed. These aspects demonstrated hypotheses H1 and H4 regarding the evolution of colon cancer incidence and mortality in the population in terms of economic development and welfare in the 25 member states analysed. The above developments are highlighted in Figure 7.

According to some authors [22], lung cancer is the leading cause of death at the European level, followed by colorectal, breast, and pancreatic cancers. Other authors [34] showed that pancreatic cancer incidence and mortality rates were influenced by patient age. The incidence rate of pancreatic cancer in the EU decreased for men aged 25–49 years and 50–64 years, while it increased for those aged 65 years and older over the period analysed.

**Figure 8:**
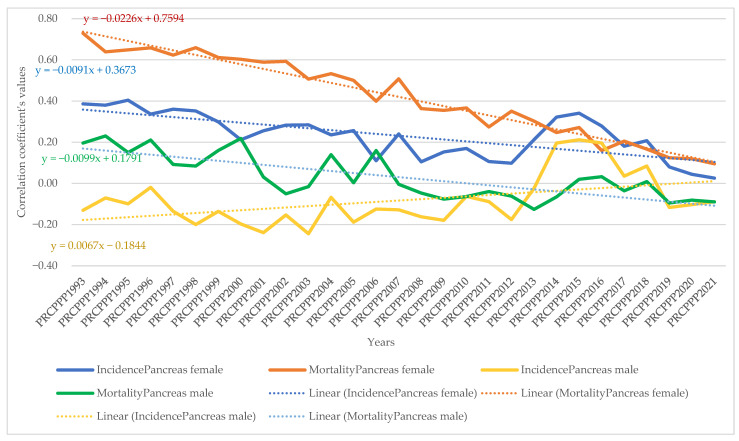
Trends in sex-standardised correlation coefficients between index and mortality rates for pancreatic cancer in relation to the dependent variable purchasing power parities (PPPs), price level indices, and real expenditures.

The evolution of pancreatic cancer incidence among the male population had been slightly unfavourable in the sense of variation between the minimum point of −24% in 2003 and the maximum point of 21% in 2015 (unfavourable evolution). Translating the variations into a polynomial regression of the second degree showed that the trend in the evolution was increasing, although there were disturbances due to the pandemic. The amplitude of the variation was at a maximum after 2015, thus destabilising the evolution of pancreatic cancer and increasing the difficulty of determining the incidence in relation to the increase in economic welfare. In the case of the female population, the evolution of incidence rates was much more favourable, decreasing from 40% in 1995 to 2% in 2021, although they were recorded in a correlation range with unfavourable positive values. In the case of mortality, the downward trend of directly proportional correlations was more accelerated, from 72% in 1993 to 9% in 2021. It was found that there was an influence of increasing economic welfare on reducing incidence and mortality rates for both women and men with pancreatic cancer. Considering the evolution of this disease and the fulminant development of the stadiality of the disease, we can state that in the case of pancreatic cancer there has been no stabilisation of the phenomenon, likely because it is more difficult to introduce prevention strategies for this type of cancer than for the other previously analysed types of cancer. These observations demonstrated hypotheses H2 and H4 of the research. The developments described above are highlighted in Figure 8.

The literature [99] showed that lung cancer ranks first in terms of incidence and mortality rates, depending on smoking prevalence and the regional human development index. According to another study (Huang et al., 2022), lung cancer incidence and mortality were based on their correlations with the human development index and social welfare. Lung cancer incidence and mortality rates tended to increase in women and decrease in men, which we also found in our analysis.

**Figure 9:**
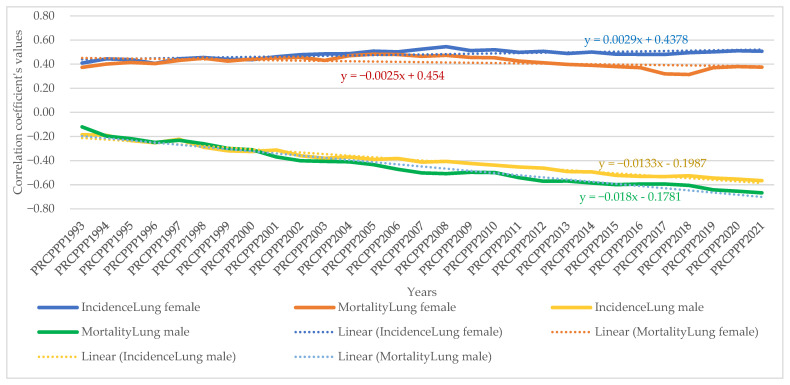
Trends in sex-standardised correlation coefficients between index and mortality rates for lung cancer in relation to the dependent variable purchasing power parities (PPPs), price level indices, and real expenditures.

Lung cancer incidence rates, as shown in the correlation table in Table 4, were inversely correlated with economic welfare growth, which was related to the strength of policies to prevent the incidence of this highly publicised disease at the EU level, especially in regards to the main cause of the disease (smoking). It can be seen from Table 4 that the minimum favourable value of the inversely proportional correlation was recorded in 1993, at −18.4%, which meant that at that time increasing economic welfare by one unit influenced the reduction of disease incidence by 0.2 units. The maximum value of the favourable negative correlation was recorded in 2021, at −56%, thus for an increase in economic welfare by one unit, the effect on disease incidence was reduced by 0.56 units. In the case of mortality, the evolution was more accelerated, with favourable negative values being recorded throughout the period analysed. The minimum negative values were related to the years 1993–1994, while the maximum values were related to the period 2019–2021. In the case of women, the incidence of the disease was unfavourably and positively correlated with the increase in economic welfare, with the trend unfavourably increasing from 40% in 1993 to 50% in 2019–2021 for the same correlated situation analysed. It was observed that in the case of lung cancer, the level of financial allocation to health did not directly influence incidence and mortality, and there was a stronger correlation between incidence and the dependent variable (level of economic welfare). These issues could be seen in terms of the decrease in the allocation process during the pandemic period, which did not influence the trend in incidence and mortality as much as the increase in economic welfare. The analysis presented on the correlations between incidence and mortality of lung cancer by sex and changes in economic welfare demonstrated hypotheses H1 and H3. The trends presented above are highlighted in Figure 9.

**Figure 10:**
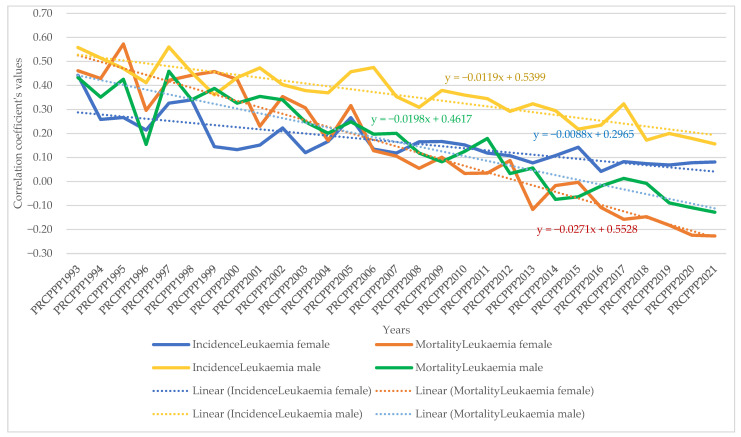
Trends in sex-standardised correlation coefficients between index and mortality rates for Leukaemia in relation to the dependent variable purchasing power parities (PPPs), price level indices, and real expenditures.

Studies in the literature highlighted the high costs of leukaemia treatment [47]. An increase in the number of men and older people affected due to risk factors such as high body mass index and smoking was noted. Other authors [48] emphasised that incidence and mortality rates of this type of cancer correlated with disability-adjusted life years and age-standardised corresponding rates. According to [49], Germany, Korea, Japan, Canada, and the UK were experiencing a rising trend in the incidence of leukaemia. In the Philippines, Ecuador, Belarus, and Thailand, leukaemia deaths were increasing. Our analysis revealed that in the case of leukaemia in the male population, there was an unfavourable correlation between the incidence of leukaemia and the increase in economic welfare. Pearson correlation coefficients were directly proportional over the period of analysis, but the trend was downward, from the maximum direct correlation value of 55.8% in 1993 to the minimum value of 15% in 2021. The mortality rates showed an acceleration of the favourable downward trend. In 2014 there was a reversal of the correlation, which until then was directly unfavourable, and which became a favourable inversely proportional correlation with a peak in 2021. Among women, there was little difference in the change in incidence compared to men, with a downward trend from 44% in 1993 to a low of 8% for the unfavourable direct correlation in 2021. As far as mortality rates were concerned, there was a reversal of the trend related to the year 2013. The range had a maximum value of 57% in 1995 and a minimum value of 22.7% in 2021. These developments validated hypotheses H2 and H4 of the research. The developments presented above are highlighted in Figure 10.

**Figure 11:**
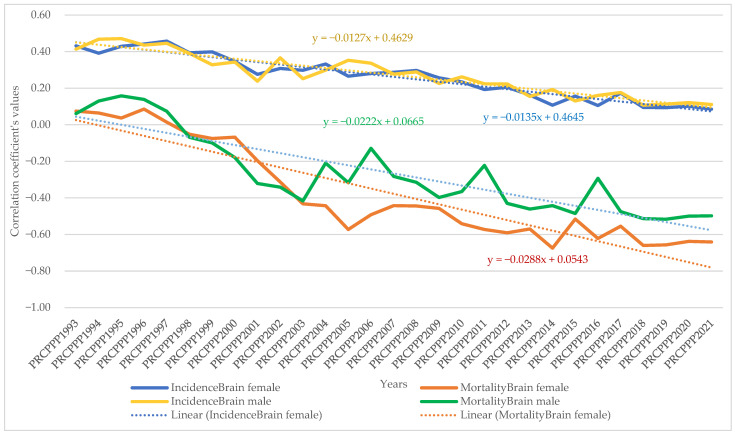
Trends in sex-standardised correlation coefficients between index and mortality rates for brain and central nervous system cancer in relation to the dependent variable purchasing power parities (PPPs), price level indices, and real expenditures.

According to the literature [100], brain and other central nervous system tumors cause high incidence and mortality rates in the US. Entities empowered to monitor and treat this type of cancer are focused on histology, age, sex, race, and anatomic site. From our point of view, the incidence rate of brain and central nervous system cancer for the male population had a positive downward trend throughout the observation period, from 47% in 1995 to 10.7% in 2018. For mortality rates, the acceleration of the downward trend was observed with an inflection point in 1998 from directly to inversely proportional correlations and quite large annual variations in the level of the correlation. The maximum value of the inverse correlation was 51.7% in 2019. During the pandemic period, the negative correlation values remained around the 505 level, with a constant decreasing trend. In the case of brain and central nervous system cancer, incidence rates for women varied in relation to the evolution of economic welfare on a downward slope ranging from a high of 45.7% (unfavourable) in 1997 to a low of 8.5% in 2021. Mortality rates had a much more favourable accelerated evolution with a change in the correlation trend in 1998 and a steeper acceleration of the slope from 2010 onwards, so that the maximum negative value of the inverse correlation was reached in 2021. These results indicated the existence of the dependence of mortality on the level of economic welfare of patients. This type of cancer has a specific need for costly palliative care for patients, thus increased economic welfare led to the strongest reduction in mortality rates of all the analysed cancers. All of these observations demonstrated hypotheses H1 and H4 of the research. The developments presented above are highlighted in Figure 11.

The analysis carried out showed that there were significant differences in the impact of increasing economic welfare on the incidence and mortality of different types of cancer according to gender, which demonstrated the need for the implementation of public health policies that succeed in limiting the disparities found in the regional dynamic cancer model. These public health policies can be:

- Additional measures to ensure early detection of early stages of lip, oral cavity, and pharyngeal, pancreatic, and brain and central nervous system cancers;
- Additional funding for lip, oral cavity, and pharyngeal cancer and lung cancer treatments to prevent mortality among women;
- Additional funding for brain and central nervous system cancer treatments to prevent mortality in men;
- Additional financial support to compensate for the costs of medicines in the treatment of various types of cancer;
- Strengthening support to provide palliative treatment for brain and central nervous system cancer and lung cancer;
- Reducing disparities in the financing of treatment costs between EU member states;
- Supporting a proactive approach to cancer by patients and their families.

## 6. Conclusions

The authors studied trends in incidence and mortality rates for lip, oral cavity, and pharyngeal, colon, pancreatic, lung, leukaemia, and brain and central nervous system cancers (Research Objective 1) and found that there were large disparities in trends in incidence and mortality rates between women and men. Overall, both incidence and mortality rates increased on average in the EU27 region, excluding Luxembourg and Cyprus, by 50% at the end of the period compared to the beginning of the period, with significant variations depending on the type of cancer, ranging from 31% for pancreatic cancer to 92% for lip, oral cavity, and pharyngeal cancer. The range of increased mortality ranged from 25% for leukaemia to 76% for pancreatic cancer (Appendix A).

The authors conducted an extensive literature review (Research Objective 2), which highlighted trends in incidence and mortality rates of different cancers at European and global levels, policies needed to improve the management of investigation and treatment of the disease, and its prevention.

In the study, the regional dynamic cancer model (Research Objective 3) was conceptualised, tested, and validated, and all working hypotheses were validated as follows. H1: There was a strong indirect connection between the level of economic development of states and mortality rates of lip, oral cavity, and pharyngeal, lung, colon, and brain and central nervous system cancers, with results being differentiated by gender. H2: There was a weak direct connection with a decreasing trend in intensity between the level of economic development and mortality rates due to leukaemia and pancreatic cancer, with the results being differentiated by gender. H3: There was a strong indirect bidirectional connection for men and a strong direct bidirectional connection for women between the level of economic development of countries and mortality rates of lung and lip, oral cavity, and pharyngeal cancers. H4: There was a weak direct connection with a decreasing trend in intensity between the level of economic development and incidence rates of leukaemia, colon, pancreatic, and brain and central nervous system cancers, with the results being differentiated by gender.

At the same time, the authors carried out staging of the dynamic regional pattern of cancer by sex and compared the effects of increasing economic welfare and health financial allocations by sex to limit the increase in incidence and mortality rates of the six cancers (Research Objective 4). This comprehensive analysis covering 25 countries and a period of 29 years (1993–2021) resulted in recommendations to implement seven public policy measures on cancer prevention and treatment and active measures to reduce regional disparities.

The study is a novel and impactful case study as it demonstrates that increasing economic well-being is a potential factor in halting the effects of cancer incidence and mortality at the population level, while the financial allocations of EU member countries’ budgets are a drawback due to large regional disparities and variability in allocations depending on vulnerabilities induced by the pandemic and economic crises.

The main limitations of the study were the lack of official data for all EU27 member states, with two member states excluded from the study, and the strong regional disparities in financial allocations, which constrained the research in terms of currency convergence and representativeness of allocations in the total national budget.

The authors aim to develop a dynamic global model of cancer based on the results obtained in the present study.

## Figures and Tables

**Table 1:** Table of indicators entered in the analysis.

Type of Cancer	Type of Indicator	Unit Measure	Symbol	
Lip, oral cavity, and pharyngeal	Incidence	Crude rate per 100,000, incidence, males and females	IncidenceLip	
	Mortality	Crude rate per 100,000, incidence, males and females	MortalityLip	
Colon	Incidence	Crude rate per 100,000, incidence, males and females	IncidenceColon	
	Mortality	Crude rate per 100,000, incidence, males and females	MortalityColon	
Pancreatic	Incidence	Crude rate per 100,000, incidence, males and females	IncidencePancreas	
	Mortality	Crude rate per 100,000, incidence, males and females	MortalityPancreas	
Lung	Incidence	Crude rate per 100,000, incidence, males and females	IncidenceLung	
	Mortality	Crude rate per 100,000, incidence, males and females	MortalityLung	
Leukaemia	Incidence	Crude rate per 100,000, incidence, males and females	IncidenceLeukaemia	
	Mortality	Crude rate per 100,000, incidence, males and females	MortalityLeukaemia	
Brain and central nervous system	Incidence	Crude rate per 100,000, incidence, males and females	IncidenceBrain	
	Mortality	Crude rate per 100,000, incidence, males and females	MortalityBrain	
	General government expenditure by function (COFOG)	Percentage of gross domestic product (GDP)	COFOG	
	Purchasing power parity (PPP), price level index, and real expenditures	Nominal expenditure per inhabitant (in euro)	PRCPPP ^1^	

^1^ dependent variable of the model, Source: Realised by authors.

**Table 2:** The values of unstandardised coefficients in the regional dynamic model of cancer.

Unstandardised Coefficients		(Constant)	COFOG	Lip		Colon		Pancreatic		Lung		Leukaemia		Brain	
Year	Gender			Incidence	Morta lity	Incidence	Morta lity	Incidence	Morta lity	Incidence	Morta lity	Incidence	Morta lity	Incidence	Morta lity
1993	male	−86.040	1.136	3.080	−8.770	2.225	−5.907	−9.906	21.875	−0.353	0.799	12.417	−10.003	4.281	−15.500
	female	19.140	0.344	4.062	−35.331	3.112	−2.769	−8.988	16.703	−0.880	0.418	6.595	−5.806	3.961	−13.540
1994	male	−124.771	1.298	3.822	−8.542	4.951	−9.119	−9.143	23.787	0.328	0.224	0.528	−7.791	6.543	−8.244
	female	−82.802	1.892	18.590	−51.174	2.413	−4.751	−8.479	16.526	−1.437	1.233	7.505	−1.428	3.110	−9.641
1995	male	−81.949	1.985	3.202	−5.972	4.783	−9.010	−10.265	17.292	0.556	−0.592	1.839	0.461	5.239	−11.036
	female	−99.790	1.558	16.638	−33.630	4.614	−7.073	−3.490	12.744	−4.978	4.101	8.809	−2.439	3.628	−14.714
1996	male	9.618	0.872	1.758	−5.059	5.700	−5.734	−12.389	18.874	−0.071	0.141	−0.988	−12.363	9.045	−11.853
	female	−56.473	1.117	22.647	−31.317	3.720	−6.850	−3.545	8.440	−4.148	4.241	5.513	−0.585	2.932	−10.783
1997	male	−94.246	1.954	3.120	−6.945	4.508	−4.683	−8.745	20.708	1.269	−0.797	−0.188	−20.494	9.530	−12.183
	female	−96.660	1.278	9.078	−33.229	2.126	−0.451	−1.749	11.208	−3.755	2.632	13.506	−21.721	5.097	−3.399
1998	male	−32.252	0.561	2.169	−7.243	5.182	−5.865	−13.616	22.988	−1.599	1.944	−1.483	−5.422	3.685	−6.985
	female	−37.969	0.557	−7.175	−10.224	2.163	1.081	−11.111	19.389	−0.563	0.514	6.965	−11.517	3.245	−6.156
1999	male	−5.952	1.129	0.715	−4.426	1.680	−1.093	−11.828	17.892	0.558	−1.087	3.525	−0.223	3.986	−12.779
	female	−26.719	1.748	−1.343	−9.035	2.177	−1.362	−9.978	20.515	2.171	−3.352	5.048	−10.480	3.825	−20.287
2000	male	−29.915	0.923	2.455	−7.747	1.563	−1.043	−8.303	20.630	−0.607	1.014	7.804	−17.363	3.555	−14.564
	female	−39.605	1.847	12.567	−41.681	2.746	−5.276	−4.936	13.145	−0.855	1.912	4.270	−2.180	2.893	−18.859
2001	male	−38.963	1.855	1.018	−3.271	−1.300	1.041	−12.073	20.943	1.592	−1.820	12.951	−8.922	1.660	−19.124
	female	18.507	1.338	16.250	−34.572	3.576	−6.774	−0.777	5.655	−1.210	2.698	−0.557	1.324	4.074	−23.876
2002	male	19.396	1.549	0.836	−3.610	1.166	−2.129	−5.670	11.174	0.743	−1.298	3.321	1.438	3.962	−16.634
	female	−16.541	1.303	−5.946	−7.025	1.714	−1.249	−9.222	15.499	3.788	−3.499	2.034	0.132	3.120	−15.125
2003	male	−36.198	1.661	1.269	−4.213	1.303	−2.315	−11.210	21.755	0.093	0.007	6.853	−11.102	4.360	−17.865
	female	−49.031	2.062	8.035	−30.333	−0.815	0.901	−2.931	9.645	2.487	−1.995	6.264	−4.302	3.934	−17.503
2004	male	−116.733	2.246	1.445	−3.340	0.876	−0.927	−4.320	12.592	0.728	−1.632	4.920	−6.637	1.442	1.171
	female	−7.967	1.699	5.265	−23.117	0.487	−1.518	−7.087	12.579	0.968	−0.703	4.178	1.804	2.561	−18.420
2005	male	42.673	1.852	−0.747	−1.919	0.545	−1.705	−5.665	10.510	1.162	−2.156	8.148	−2.974	0.582	−12.209
	female	104.668	0.320	7.684	−29.843	1.935	−3.953	−4.446	6.146	1.143	0.608	−4.073	6.664	1.383	−14.167
2006	male	3.442	1.680	0.197	−2.906	0.275	−0.783	−6.154	11.322	0.831	−1.545	6.360	−2.124	1.879	−10.043
	female	116.613	−0.145	16.017	−29.256	0.054	−5.638	0.757	1.945	−1.081	3.344	1.585	4.492	2.825	−17.164
2007	male	−5.330	2.288	3.011	−3.088	2.924	−8.204	−8.741	11.896	−0.260	0.814	−2.529	−0.351	6.771	−13.588
	female	43.679	0.768	12.990	−13.262	1.518	−6.431	−5.110	8.034	−0.780	1.245	0.191	6.034	2.190	−13.623
2008	male	−81.044	2.888	1.465	−3.861	1.118	−3.415	−5.868	13.455	0.656	−0.273	6.476	−16.566	3.226	−7.560
	female	66.250	0.739	8.846	−23.246	1.930	−6.707	−3.930	5.942	−1.588	2.587	2.009	7.477	3.394	−17.845
2009	male	19.698	1.888	1.396	−3.147	1.468	−4.220	−4.620	9.338	−0.041	−0.033	3.104	−4.412	2.086	−10.465
	female	82.513	0.026	3.904	−50.314	−0.765	2.024	−0.414	8.013	−0.310	1.906	4.430	0.149	3.532	−22.820
2010	male	56.652	0.510	1.419	−4.605	1.471	−3.714	−6.695	10.964	0.457	−0.530	1.867	5.245	3.829	−17.403
	female	25.205	2.198	3.593	−21.986	−2.084	1.169	−3.381	8.552	3.584	−3.307	2.398	−3.721	0.798	−10.002
2011	male	51.611	2.969	−1.674	−1.844	−0.111	1.111	−1.123	1.990	1.907	−3.392	4.723	−2.753	0.090	−4.840
	female	−42.105	2.831	7.311	−11.801	0.679	−3.300	−2.632	4.691	0.091	0.539	3.080	−4.113	0.970	−6.995
2012	male	48.878	3.586	−1.720	−2.030	−0.744	1.655	−2.094	4.542	1.643	−2.893	3.161	−2.328	−0.199	−7.405
	female	54.287	2.613	0.614	−23.699	−1.136	0.132	−3.943	7.208	3.670	−3.014	2.157	−5.702	0.590	−10.592
2013	male	70.774	1.185	1.591	−3.096	1.231	−3.246	−1.247	3.379	−0.611	0.286	2.614	−2.551	1.445	−7.872
	female	96.762	0.582	9.179	−40.160	0.384	−2.880	1.487	−0.780	−1.027	2.897	2.821	3.826	0.901	−12.936
2014	male	93.700	2.509	−0.177	−3.681	−0.674	1.009	4.355	−2.388	0.167	−0.759	4.871	−8.760	0.258	−6.890
	female	45.717	2.882	2.639	−9.473	0.107	−4.089	−3.350	2.326	2.789	−2.306	3.025	−0.865	0.269	−9.693
2015	male	113.616	1.537	−0.038	−2.571	1.122	−2.813	−1.202	0.852	−0.196	−0.749	2.187	3.739	0.845	−7.351
	female	79.077	1.045	4.542	−18.028	0.598	−5.639	−0.582	2.338	−0.191	1.444	3.393	1.661	1.095	−10.155
2016	male	19.049	2.854	−1.088	−1.149	0.785	−1.216	−1.027	2.351	−0.240	−1.325	4.311	0.792	−0.569	−0.994
	female	82.521	1.354	4.609	−28.109	0.362	−3.424	−0.852	2.799	0.447	0.824	2.745	−0.917	1.135	−10.526
2017	male	120.242	1.713	−0.020	−1.248	1.306	−3.504	−2.945	1.190	0.325	−1.058	1.403	5.343	1.721	−11.735
	female	126.934	0.748	4.403	−26.365	−0.169	−0.279	−2.484	5.123	1.253	−0.745	3.002	−5.392	1.610	−15.938
2018	male	147.363	0.796	0.838	−1.763	0.757	−2.592	−2.690	2.310	0.240	−0.906	−1.058	7.665	2.015	−12.197
	female	117.359	0.041	4.355	−25.694	−0.225	−2.066	0.505	2.524	0.435	0.649	3.003	−1.844	1.371	−10.418
2019	male	137.066	0.985	−0.011	−1.879	0.127	0.095	−1.506	1.125	0.636	−1.727	1.681	3.233	0.881	−8.689
	female	96.788	0.583	5.191	−4.548	−0.075	−3.179	−0.931	0.366	1.125	−0.455	5.054	−4.998	0.698	−7.639
2020	male	117.468	1.161	0.853	−1.677	1.035	−3.135	−1.438	0.934	0.207	−0.527	0.682	0.023	1.153	−7.555
	female	84.225	1.569	0.009	−6.108	0.967	−4.732	−2.706	2.450	1.186	−0.788	2.742	−0.025	0.727	−9.642
2021	male	122.991	1.457	0.381	−1.132	1.034	−2.947	−1.925	0.056	0.271	−0.793	−0.070	2.973	1.191	−6.258
	female	105.850	1.439	−0.682	−6.697	1.212	−4.981	−2.690	2.016	1.014	−0.633	1.872	2.332	0.727	−10.126

Source: Realised by authors using [1,93,94,95,96,97].

**Table 3:** Representation of ANOVA test results ^1^.

ANOVA		Female					Male						Compared Models Male vs. Female		
		Sum of Squares	df	Mean Square	F	Sig.	Sum of Squares	df	Mean Square	F	Sig.	Sum of Squares (M/F%)	F (M/F%)	H_0_ Male (a < 0.01)	H_0_ Female (a < 0.01)
1993	Regression	82,959.652	13	6381.5117	3.8758706	0.015	87,628.094	13	6740.6226	5.5157508	0.004	105.63%	142.31%	R.NHyp ^2^	
	Residual	18,111.19	11	1646.4718			13,442.748	11	1222.068			74.22%			
1994	Regression	85,331.736	13	6563.9797	7.0726407	0.001	79,472.486	13	6113.2682	4.1850501	0.012	93.13%	59.17%		R.NHyp
	Residual	10,208.885	11	928.08046			16,068.135	11	1460.7396			157.39%			
1995	Regression	78,623.689	13	6047.9761	5.5389873	0.004	70,936.577	13	5456.6598	3.0471869	0.036	90.22%	55.01%		R.NHyp
	Residual	12,010.812	11	1091.892			19,697.925	11	1790.7204			164.00%			
1996	Regression	77,477.752	13	5959.8271	7.4608524	0.001	77,365.488	13	5951.1914	7.3560596	0.001	99.86%	98.60%	R.NHyp	R.NHyp
	Residual	8786.9447	11	798.81315			8899.2081	11	809.01892			101.28%			
1997	Regression	66,849.014	13	5142.2319	3.6458185	0.019	70,363.701	13	5412.5924	4.9614504	0.006	105.26%	136.09%	R.NHyp	
	Residual	15,514.911	11	1410.4465			12,000.224	11	1090.9295			77.35%			
1998	Regression	65,278.451	13	5021.4193	4.0623023	0.013	68,922.472	13	5301.7286	5.8593821	0.003	105.58%	144.24%	R.NHyp	
	Residual	13,597.12	11	1236.1018			9953.0997	11	904.82725			73.20%			
1999	Regression	66,181.039	13	5090.8492	5.8577214	0.003	62,412.215	13	4800.9396	3.9621392	0.014	94.31%	67.64%		R.NHyp
	Residual	9559.9188	11	869.08353			13,328.743	11	1211.7039			139.42%			
2000	Regression	66,701.862	13	5130.9125	9.0762499	0	64,208.128	13	4939.0868	6.2361025	0.002	96.26%	68.71%	R.NHyp	R.NHyp
	Residual	6218.4314	11	565.31195			8712.1652	11	792.01501			140.10%			
2001	Regression	65,728.255	13	5056.0196	11.965925	0	64,034.994	13	4925.7688	8.5447449	0.001	97.42%	71.41%	R.NHyp	R.NHyp
	Residual	4647.8828	11	422.5348			6341.1438	11	576.46762			136.43%			
2002	Regression	58,054.951	13	4465.7654	4.9007422	0.006	56,982.771	13	4383.2901	4.3454258	0.01	98.15%	88.67%		R.NHyp
	Residual	10,023.669	11	911.24268			11,095.849	11	1008.7136			110.70%			
2003	Regression	60,543.935	13	4657.2257	9.3988606	0	58,714.191	13	4516.4762	6.8240186	0.002	96.98%	72.60%	R.NHyp	R.NHyp
	Residual	5450.6057	11	495.50961			7280.3493	11	661.84993			133.57%			
2004	Regression	58,072.298	13	4467.0999	8.1452879	0.001	50,267.019	13	3866.6938	3.0736876	0.035	86.56%	37.74%		R.NHyp
	Residual	6032.7025	11	548.4275			13,837.982	11	1257.9983			229.38%			
2005	Regression	54,862.385	13	4220.1834	6.1695484	0.002	54,154.671	13	4165.7439	5.5664079	0.004	98.71%	90.22%	R.NHyp	R.NHyp
	Residual	7524.3786	11	684.03442			8232.0922	11	748.37202			109.41%			
2006	Regression	56,156.079	13	4319.6984	10.176813	0	54,602.703	13	4200.2079	7.4250505	0.001	97.23%	72.96%	R.NHyp	R.NHyp
	Residual	4669.1123	11	424.46476			6222.488	11	565.68073			133.27%			
2007	Regression	55,005.414	13	4231.1857	10.596327	0	51,868.907	13	3989.9159	5.8294274	0.003	94.30%	55.01%	R.NHyp	R.NHyp
	Residual	4392.3753	11	399.30684			7528.8827	11	684.44388			171.41%			
2008	Regression	52,120.287	13	4009.2528	7.3796384	0.001	52,762.664	13	4058.6665	8.3703202	0.001	101.23%	113.42%	R.NHyp	R.NHyp
	Residual	5976.1439	11	543.28581			5333.7663	11	484.88784			89.25%			
2009	Regression	50,326.305	13	3871.2542	6.4737687	0.002	48,474.149	13	3728.7807	4.8655183	0.006	96.32%	75.16%	R.NHyp	R.NHyp
	Residual	6577.899	11	597.99082			8430.0552	11	766.36865			128.16%			
2010	Regression	48,120.658	13	3701.589	5.290958	0.005	50,877.153	13	3913.6272	8.716007	0.001	105.73%	164.73%	R.NHyp	R.NHyp
	Residual	7695.6725	11	699.60659			4939.1767	11	449.01606			64.18%			
2011	Regression	47,640.891	13	3664.6839	5.6170233	0.004	47831.947	13	3679.3805	5.7937905	0.003	100.40%	103.15%	R.NHyp	R.NHyp
	Residual	7176.6701	11	652.42456			6985.6143	11	635.05585			97.34%			
2012	Regression	48,296.923	13	3715.1479	8.7832306	0	49,038.244	13	3772.1726	10.608233	0	101.53%	120.78%	R.NHyp	R.NHyp
	Residual	4652.8014	11	422.98194			3911.4806	11	355.58915			84.07%			
2013	Regression	48,929.908	13	3763.839	8.5112537	0.001	43,004.683	13	3308.0525	3.3725495	0.026	87.89%	39.62%		R.NHyp
	Residual	4864.4102	11	442.21911			10,789.635	11	980.87589			221.81%			
2014	Regression	46,327.916	13	3563.6858	6.0676754	0.003	44,895.055	13	3453.4658	4.8126349	0.007	96.91%	79.32%	R.NHyp	R.NHyp
	Residual	6460.5539	11	587.32309			7893.4148	11	717.58316			122.18%			
2015	Regression	44,170.079	13	3397.6984	5.2517248	0.005	43,805.729	13	3369.6715	4.9547373	0.006	99.18%	94.34%	R.NHyp	R.NHyp
	Residual	7116.649	11	646.96809			7480.9992	11	680.09084			105.12%			
2016	Regression	46,061.312	13	3543.1778	9.5686186	0	41570.068	13	3197.6975	4.1070554	0.012	90.25%	42.92%		R.NHyp
	Residual	4073.2062	11	370.29147			8564.4504	11	778.5864			210.26%			
2017	Regression	42,092.459	13	3237.8815	7.6141015	0.001	41,718.972	13	3209.1517	6.988549	0.001	99.11%	91.78%	R.NHyp	R.NHyp
	Residual	4677.728	11	425.248			5051.2157	11	459.20143			107.98%			
2018	Regression	39,670.453	13	3051.5733	7.9672801	0.001	37,460.119	13	2881.5476	4.9345567	0.006	94.43%	61.94%	R.NHyp	R.NHyp
	Residual	4213.1451	11	383.01319			6423.4794	11	583.95267			152.46%			
2019	Regression	36,490.28	13	2806.9446	6.1589549	0.002	33,311.988	13	2562.4606	3.4409961	0.024	91.29%	55.87%		R.NHyp
	Residual	5013.2516	11	455.75015			8191.5428	11	744.68571			163.40%			
2020	Regression	36,152.692	13	2780.9763	4.6221106	0.008	34,349.946	13	2642.3035	3.4514915	0.023	95.01%	74.67%		R.NHyp
	Residual	6618.3487	11	601.66806			8421.0953	11	765.55411			127.24%			
2021	Regression	35,304.427	13	2715.7251	5.0810538	0.005	33,404.997	13	2569.6152	3.6337311	0.02	94.62%	71.52%		R.NHyp
	Residual	5879.2875	11	534.48069			7778.7173	11	707.15612			132.31%			

^1^ Realised by authors using IBM-SPSS25 software. Dependent Variable: PRCPPP (year). Predictors: (Constant), COFOG (year), IncidenceLip (year), MortalityLip (year), IncidenceColon (year), MortalityColon (year), MortalityPancreas (year), IncidencePancreas (year), IncidenceLung (year), MortalityLung (year), MortalityLeukaemia (year), IncidenceLeukaemia (year), MortalityBrain (year), IncidenceBrain (year). ^2^ R.NHyp-reject null hypothesis.

**Table 4:** Pearson coefficient analysis ^1^.

Pearson Correlation	Gender	COFOG	Lip		Colon		Pancreatic		Lung		Leukaemia		Brain	
			Incidence	Mortality	Incidence	Mortality	Incidence	Mortality	Incidence	Mortality	Incidence	Mortality	Incidence	Mortality
PRCPPP1993	M	0.473	−0.039	−0.327	0.562	0.467	−0.131	0.196	−0.184	−0.120	0.558	0.433	0.413	0.061
	F	0.473	0.412	0.255	0.616	0.535	0.386	0.729	0.409	0.374	0.442	0.461	0.431	0.074
PRCPPP1994	M	0.488	0.016	−0.344	0.533	0.415	−0.071	0.230	−0.191	−0.197	0.514	0.351	0.468	0.129
	F	0.488	0.470	0.185	0.586	0.511	0.380	0.639	0.444	0.401	0.259	0.429	0.391	0.064
PRCPPP1995	M	0.503	0.007	−0.379	0.509	0.441	−0.099	0.150	−0.233	−0.218	0.471	0.426	0.471	0.158
	F	0.503	0.506	0.191	0.615	0.537	0.404	0.648	0.438	0.414	0.267	0.573	0.429	0.037
PRCPPP1996	M	0.518	−0.005	−0.409	0.491	0.419	−0.020	0.210	−0.252	−0.250	0.412	0.154	0.435	0.138
	F	0.518	0.752	0.192	0.569	0.496	0.336	0.658	0.407	0.405	0.214	0.297	0.441	0.086
PRCPPP1997	M	0.533	−0.079	−0.399	0.489	0.383	−0.136	0.092	−0.223	−0.231	0.560	0.459	0.445	0.074
	F	0.533	0.347	0.234	0.560	0.515	0.360	0.623	0.444	0.431	0.326	0.421	0.457	0.014
PRCPPP1998	M	0.547	−0.061	−0.418	0.417	0.324	−0.200	0.084	−0.288	−0.260	0.454	0.340	0.390	−0.068
	F	0.547	0.269	0.160	0.565	0.503	0.352	0.659	0.455	0.449	0.340	0.442	0.394	−0.052
PRCPPP1999	M	0.561	−0.152	−0.458	0.410	0.335	−0.136	0.160	−0.319	−0.298	0.361	0.388	0.328	−0.100
	F	0.561	0.406	0.157	0.557	0.484	0.299	0.611	0.440	0.425	0.145	0.458	0.398	−0.076
PRCPPP2000	M	0.575	−0.123	−0.433	0.320	0.243	−0.197	0.218	−0.325	−0.309	0.433	0.326	0.343	−0.179
	F	0.575	0.490	0.221	0.520	0.471	0.211	0.603	0.437	0.442	0.133	0.426	0.346	−0.068
PRCPPP2001	M	0.588	−0.141	−0.485	0.300	0.221	−0.239	0.031	−0.312	−0.369	0.473	0.354	0.238	−0.321
	F	0.588	0.443	0.013	0.495	0.467	0.255	0.588	0.461	0.452	0.152	0.230	0.275	−0.197
PRCPPP2002	M	0.602	−0.116	−0.493	0.255	0.108	−0.153	−0.051	−0.360	−0.401	0.403	0.340	0.365	−0.341
	F	0.602	0.434	0.098	0.498	0.401	0.283	0.592	0.479	0.456	0.223	0.354	0.308	−0.313
PRCPPP2003	M	0.613	−0.105	−0.478	0.286	0.094	−0.244	−0.016	−0.380	−0.407	0.379	0.248	0.252	−0.416
	F	0.613	0.438	0.152	0.454	0.381	0.284	0.506	0.485	0.431	0.120	0.307	0.298	−0.432
PRCPPP2004	M	0.625	−0.152	−0.499	0.268	0.060	−0.068	0.139	−0.370	−0.410	0.369	0.201	0.299	−0.210
	F	0.625	0.502	0.078	0.411	0.296	0.235	0.533	0.487	0.470	0.167	0.170	0.332	−0.443
PRCPPP2005	M	0.635	−0.169	−0.561	0.208	−0.045	−0.188	0.003	−0.391	−0.434	0.457	0.249	0.352	−0.317
	F	0.635	0.539	−0.104	0.522	0.318	0.256	0.500	0.508	0.483	0.266	0.316	0.265	−0.573
PRCPPP2006	M	0.643	−0.082	−0.583	0.251	−0.047	−0.125	0.159	−0.382	−0.472	0.475	0.197	0.336	−0.129
	F	0.643	0.675	−0.022	0.422	0.239	0.110	0.399	0.502	0.480	0.135	0.128	0.280	−0.492
PRCPPP2007	M	0.650	−0.115	−0.602	0.226	−0.168	−0.129	−0.004	−0.413	−0.502	0.354	0.201	0.277	−0.283
	F	0.650	0.654	−0.026	0.435	0.142	0.240	0.507	0.525	0.465	0.118	0.106	0.287	−0.442
PRCPPP2008	M	0.654	−0.054	−0.586	0.197	−0.166	−0.162	−0.047	−0.406	−0.508	0.309	0.118	0.289	−0.315
	F	0.654	0.513	−0.005	0.374	0.134	0.105	0.363	0.545	0.473	0.165	0.055	0.297	−0.444
PRCPPP2009	M	0.655	−0.044	−0.635	0.147	−0.249	−0.180	−0.077	−0.424	−0.497	0.379	0.082	0.226	−0.398
	F	0.655	0.464	−0.029	0.422	0.133	0.152	0.354	0.512	0.456	0.167	0.101	0.256	−0.457
PRCPPP2010	M	0.653	−0.091	−0.596	0.094	−0.247	−0.063	−0.064	−0.438	−0.499	0.360	0.125	0.262	−0.366
	F	0.653	0.617	−0.161	0.343	0.053	0.169	0.366	0.519	0.453	0.152	0.034	0.234	−0.541
PRCPPP2011	M	0.646	−0.075	−0.635	0.084	−0.290	−0.089	−0.040	−0.453	−0.542	0.345	0.179	0.224	−0.223
	F	0.646	0.683	−0.061	0.362	0.032	0.106	0.274	0.498	0.426	0.118	0.035	0.193	−0.573
PRCPPP2012	M	0.690	0.038	−0.660	0.062	−0.348	−0.176	−0.062	−0.462	−0.571	0.292	0.033	0.223	−0.430
	F	0.690	0.516	−0.110	0.277	−0.134	0.098	0.350	0.506	0.411	0.108	0.087	0.205	−0.591
PRCPPP2013	M	0.537	−0.003	−0.642	0.022	−0.424	−0.024	−0.126	−0.491	−0.569	0.323	0.056	0.155	−0.461
	F	0.537	0.654	−0.116	0.280	−0.134	0.212	0.302	0.488	0.398	0.077	−0.117	0.162	−0.570
PRCPPP2014	M	0.581	0.045	−0.667	0.068	−0.408	0.196	−0.066	−0.494	−0.586	0.295	−0.075	0.192	−0.442
	F	0.581	0.771	−0.174	0.267	−0.208	0.322	0.247	0.500	0.390	0.108	−0.017	0.107	−0.674
PRCPPP2015	M	0.459	−0.016	−0.664	0.044	−0.452	0.212	0.020	−0.522	−0.599	0.218	−0.064	0.130	−0.485
	F	0.459	0.770	−0.108	0.257	−0.302	0.341	0.271	0.482	0.380	0.143	−0.003	0.156	−0.516
PRCPPP2016	M	0.540	0.070	−0.622	0.007	−0.430	0.196	0.032	−0.531	−0.594	0.235	−0.019	0.160	−0.293
	F	0.540	0.751	−0.239	0.232	−0.260	0.278	0.160	0.482	0.371	0.042	−0.108	0.105	−0.622
PRCPPP2017	M	0.510	0.015	−0.655	−0.046	−0.513	0.035	−0.036	−0.533	−0.594	0.324	0.013	0.176	−0.475
	F	0.510	0.677	−0.187	0.191	−0.272	0.180	0.205	0.480	0.319	0.083	−0.157	0.173	−0.555
PRCPPP2018	M	0.470	0.058	−0.668	−0.044	−0.534	0.084	0.008	−0.526	−0.605	0.173	−0.008	0.107	−0.513
	F	0.470	0.748	−0.189	0.124	−0.372	0.207	0.166	0.495	0.314	0.074	−0.147	0.094	−0.660
PRCPPP2019	M	0.436	−0.041	−0.618	−0.108	−0.420	−0.117	−0.095	−0.545	−0.642	0.200	−0.089	0.113	−0.517
	F	0.436	0.630	−0.179	0.020	−0.167	0.080	0.124	0.502	0.371	0.069	−0.182	0.093	−0.657
PRCPPP2020	M	0.303	0.005	−0.627	−0.110	−0.573	−0.103	−0.081	−0.554	−0.654	0.179	−0.109	0.121	−0.500
	F	0.303	0.580	−0.092	0.085	−0.453	0.044	0.119	0.511	0.380	0.078	−0.224	0.101	−0.638
PRCPPP2021	M	0.324	0.022	−0.609	−0.127	−0.591	−0.089	−0.090	−0.566	−0.667	0.157	−0.128	0.110	−0.498
	F	0.324	0.528	−0.118	0.065	−0.481	0.026	0.095	0.507	0.375	0.081	−0.227	0.085	−0.641
Min	M	0.303	−0.169	−0.668	−0.127	−0.591	−0.244	−0.126	−0.566	−0.667	0.157	−0.128	0.107	−0.517
	F	0.303	0.269	−0.239	0.020	−0.481	0.026	0.095	0.407	0.314	0.042	−0.227	0.085	−0.674
Max	M	0.690	0.070	−0.327	0.562	0.467	0.212	0.230	−0.184	−0.120	0.560	0.459	0.471	0.158
	F	0.690	0.771	0.255	0.616	0.537	0.404	0.729	0.545	0.483	0.442	0.573	0.457	0.086
Std Dev	M	0.096	0.069	0.109	0.208	0.358	0.123	0.109	0.115	0.157	0.113	0.181	0.115	0.216
	F	0.096	0.136	0.153	0.175	0.343	0.109	0.196	0.034	0.045	0.094	0.240	0.119	0.266
Average	M	0.552	−0.048	−0.543	0.201	−0.083	−0.083	0.030	−0.399	−0.449	0.361	0.165	0.272	−0.266
	F	0.552	0.560	0.001	0.384	0.133	0.231	0.420	0.481	0.417	0.165	0.146	0.262	−0.378
Amplitude	M	0.387	0.239	0.342	0.689	1.058	0.456	0.357	0.382	0.546	0.404	0.587	0.364	0.674
	F	0.387	0.502	0.494	0.596	1.018	0.378	0.634	0.139	0.169	0.400	0.800	0.372	0.760

^1^ Realised by authors using IBM-SPSS25 software.

## Data Availability

Not applicable.

## Appendix A

**Table A1:** Descriptive statistics of model indicators in the first and last year of analysis.

Indicator	Females						Males						Total					
	Mean	Median	Std. Err of Mean	Min	Max	Std. Deviation	Mean	Median	Std. Err of Mean	Min	Max	Std. Deviation	Mean	Median	Std. Err of Mean	Min	Max	Std. Deviation
IncidenceLip1993 *	4.73	4.50	0.32	2.50	8.10	1.58	18.29	16.90	1.72	7.50	43.30	8.60	11.51	7.65	1.30	2.50	43.30	9.19
IncidenceLip2021 *	8.88	8.90	0.58	4.30	15.10	2.89	23.11	21.30	1.89	10.80	57.10	9.44	16.00	14.65	1.41	4.30	57.10	9.97
MortalityLip1993 *	1.96	1.90	0.12	1.00	4.10	0.61	9.34	8.30	1.00	3.10	24.20	5.00	5.65	3.15	0.73	1.00	24.20	5.13
MortalityLip2021 *	2.97	2.90	0.18	1.20	6.30	0.91	10.81	8.50	1.25	4.30	24.80	6.23	6.89	4.35	0.84	1.20	24.80	5.92
IncidenceColon1993 *	27.40	24.20	2.25	11.20	54.60	11.26	26.87	26.20	2.21	12.10	53.30	11.04	27.13	25.90	1.56	11.20	54.60	11.04
IncidenceColon2021 *	42.53	41.10	2.20	20.10	66.20	10.99	52.41	53.00	2.89	25.40	78.00	14.44	47.47	45.55	1.93	20.10	78.00	13.64
MortalityColon1993 *	16.82	16.50	1.32	7.10	29.90	6.60	16.57	16.40	1.15	7.60	25.60	5.77	16.70	16.45	0.87	7.10	29.90	6.13
MortalityColon2021 *	20.00	20.60	0.97	6.30	30.00	4.84	24.98	23.40	1.57	8.20	44.40	7.86	22.49	21.10	0.98	6.30	44.40	6.93
IncidencePancreas1993 *	10.35	9.50	0.63	5.30	15.30	3.16	12.07	12.20	0.51	8.20	17.20	2.56	11.21	11.65	0.42	5.30	17.20	2.98
IncidencePancreas2021 *	15.59	14.70	1.01	9.00	29.40	5.06	16.62	15.40	0.94	9.30	26.90	4.69	16.11	15.30	0.69	9.00	29.40	4.85
MortalityPancreas1993 *	10.24	10.20	0.69	4.30	18.20	3.47	12.02	11.90	0.45	8.80	17.00	2.26	11.13	10.90	0.43	4.30	18.20	3.03
MortalityPancreas2021 *	18.16	18.10	0.67	10.60	22.70	3.36	19.11	19.20	0.57	11.50	23.70	2.84	18.64	19.15	0.44	10.60	23.70	3.12
IncidenceLung1993 *	17.66	15.40	1.85	7.10	47.60	9.24	79.80	81.60	4.24	39.50	117.40	21.18	48.73	40.70	4.99	7.10	117.40	35.31
IncidenceLung2021 *	43.47	42.20	3.88	19.70	93.60	19.38	86.20	85.50	4.33	40.70	128.90	21.64	64.83	62.70	4.19	19.70	128.90	29.65
MortalityLung1993 *	16.70	13.60	1.71	7.20	46.50	8.55	75.06	73.90	3.43	41.30	115.00	17.17	45.88	42.55	4.58	7.20	115.00	32.39
MortalityLung2021 *	34.96	34.10	2.88	17.70	72.30	14.40	74.18	73.00	4.05	37.20	117.90	20.24	54.57	53.65	3.73	17.70	117.90	26.35
IncidenceLeukaemia1993 *	8.97	8.80	0.48	3.00	14.40	2.42	11.06	11.50	0.61	4.40	17.80	3.04	10.02	9.75	0.41	3.00	17.80	2.92
IncidenceLeukaemia2021 *	10.04	10.50	0.74	5.30	17.10	3.72	17.13	16.70	0.72	9.90	28.00	3.60	13.59	14.20	0.72	5.30	28.00	5.09
MortalityLeukaemia1993 *	6.25	6.30	0.29	3.40	8.70	1.47	8.02	8.10	0.31	5.00	10.80	1.53	7.14	7.10	0.25	3.40	10.80	1.73
MortalityLeukaemia2021 *	7.59	7.40	0.32	4.00	10.20	1.62	9.78	9.60	0.37	6.60	14.50	1.86	8.68	8.85	0.29	4.00	14.50	2.05
IncidenceBrain1993 *	10.29	6.60	1.21	3.40	23.00	6.07	11.40	9.20	1.25	4.60	27.80	6.23	10.84	9.20	0.86	3.40	27.80	6.11
IncidenceBrain2021 *	17.37	13.30	2.20	7.00	45.40	10.98	15.58	14.00	1.35	9.30	32.50	6.73	16.47	13.55	1.28	7.00	45.40	9.06
MortalityBrain1993 *	4.83	4.80	0.19	3.10	7.00	0.95	6.12	5.90	0.26	4.20	10.20	1.28	5.47	5.30	0.18	3.10	10.20	1.29
MortalityBrain2021 *	7.10	6.80	0.39	3.80	10.70	1.94	8.82	8.30	0.35	6.20	12.70	1.77	7.96	7.90	0.29	3.80	12.70	2.03
COFOG1993 ***	49.39	47.00	3.01	27.30	83.80	15.03	49.39	47.00	3.01	27.30	83.80	15.03	49.39	47.00	2.10	27.30	83.80	14.87
COFOG2021 ***	44.68	44.10	1.53	19.80	57.90	7.63	44.68	44.10	1.53	19.80	57.90	7.63	44.68	44.10	1.07	19.80	57.90	7.55
PRCPPP1993 **	92.57	75.99	12.98	10.90	265.47	64.89	92.57	75.99	12.98	10.90	265.47	64.89	92.57	75.99	9.08	10.90	265.47	64.23
PRCPPP2021 **	90.08	74.57	8.28	33.50	171.83	41.42	90.08	74.57	8.28	33.50	171.83	41.42	90.08	74.57	5.80	33.50	171.83	41.00

* Crude rate per 100 000, incidence, males and females; ** Percentage of gross domestic product (GDP); *** Nominal expenditure per inhabitant (in euros).
